# The Impact of Soy Isoflavones on MCF-7 and MDA-MB-231 Breast Cancer Cells Using a Global Metabolomic Approach

**DOI:** 10.3390/ijms17091443

**Published:** 2016-08-31

**Authors:** Alina Uifălean, Stefanie Schneider, Philipp Gierok, Corina Ionescu, Cristina Adela Iuga, Michael Lalk

**Affiliations:** 1 Department of Pharmaceutical Analysis, Faculty of Pharmacy, “Iuliu Hațieganu” University of Medicine and Pharmacy, Louis Pasteur Street 6, Cluj-Napoca 400349, Romania; alina.uifalean@umfcluj.ro; 2 Institute of Biochemistry, Ernst-Moritz-Arndt-University, Felix-Hausdorff Street 4, Greifswald 17487, Germany; schneids42@uni-greifswald.de (S.S.); gierokp47@uni-greifswald.de (P.G.); lalk@uni-greifswald.de (M.L.); 3 Department of Pharmaceutical Biochemistry and Clinical Laboratory, Faculty of Pharmacy, “Iuliu Hațieganu” University of Medicine and Pharmacy, Louis Pasteur Street 6, Cluj-Napoca 400349, Romania; corina.ionescu@umfcluj.ro; 4 MedFuture Research Center for Advanced Medicine, “Iuliu Hațieganu” University of Medicine and Pharmacy, Louis Pasteur Street 4-6, Gh. Marinescu Street 23, Cluj-Napoca 400349, Romania

**Keywords:** soy isoflavones, breast cancer, metabolomics, MTT assay, ^1^H-NMR, GC-MS, glucose uptake, glutamine uptake

## Abstract

Despite substantial research, the understanding of the chemopreventive mechanisms of soy isoflavones remains challenging. Promising tools, such as metabolomics, can provide now a deeper insight into their biochemical mechanisms. The purpose of this study was to offer a comprehensive assessment of the metabolic alterations induced by genistein, daidzein and a soy seed extract on estrogen responsive (MCF-7) and estrogen non-responsive breast cancer cells (MDA-MB-231), using a global metabolomic approach. The 3-(4,5-dimethylthiazol-2-yl)-2,5-diphenyltetrazolium bromide (MTT) assay showed that all test compounds induced a biphasic effect on MCF-7 cells and only a dose-dependent inhibitory effect on MDA-MB-231 cells. Proton nuclear magnetic resonance (^1^H-NMR) profiling of extracellular metabolites and gas chromatography-mass spectrometry (GC-MS) profiling of intracellular metabolites confirmed that all test compounds shared similar metabolic mechanisms. Exposing MCF-7 cells to stimulatory concentrations of isoflavones led to increased intracellular levels of 6-phosphogluconate and ribose 5-phosphate, suggesting a possible upregulation of the pentose phosphate pathway. After exposure to inhibitory doses of isoflavones, a significant decrease in glucose uptake was observed, especially for MCF-7 cells. In MDA-MB-231 cells, the glutamine uptake was significantly restricted, leading to alterations in protein biosynthesis. Understanding the metabolomic alterations of isoflavones represents a step forward in considering soy and soy derivates as functional foods in breast cancer chemoprevention.

## 1. Introduction

In the USA, breast cancer is the leading cause of cancer death in women aged 20–59 years and it is expected to account for 29% of all new cancer diagnoses in women in 2016 [1]. Advances in breast cancer diagnostics and treatment are hampered by the metabolic heterogeneity of the disease, metabolic reprogramming being one of the hallmarks of tumor cells [2]. Therefore, targeting tumor metabolism would represent a rational strategy to improve cancer therapeutics.

So far, natural compounds or naturally-derived compounds have served as the main cancer therapeutics, representing over 60% of the available anti-cancer agents [3]. Apparently, nature provides a wide range of bioactive compounds with tremendous potential in chemoprevention and chemotherapy. Moreover, many of these compounds are part of the daily diet, creating the opportunity to use food as an effective prevention strategy, especially in the early stages of the disease.

One example is soy (*Glycine max*) isoflavones, extensively studied over the past 20 years. The cytotoxic potential of soy isoflavones has been explored mainly in breast cancers, where the dose-dependent effects of soy isoflavones have raised much controversy [4]. Although gene and protein expression have been profiled in breast cancer cells after isoflavone exposure [5,6], little is known regarding the metabolic alterations that characterize their effects. As the proteome and genome respond to nutrients or stress, long after the cell metabolome, only metabolomics can offer an accurate snapshot of the metabolic status.

To our knowledge, a single metabolomic study on soy isoflavones has been made so far, comparing the metabolic profiles of a non-tumorigenic epithelial breast cell line and a breast cancer cell line exposed to 17-β-estradiol, genistein or a native root flax extract. Metabolic profiling via gas chromatography-mass spectrometry (GC-MS) revealed significant changes in the sphingolipid metabolism, isoflavone treatment influencing the expression level of the sphingosine metabolizing enzymes [7]. An extracellular and intracellular metabolomic approach, using both estrogen responsive and non-responsive breast cancer cells exposed to different isoflavones, at different concentrations, would contribute to the current knowledge, offering a comprehensive assessment of the metabolic alterations induced by isoflavones in breast cancer cells.

For this purpose, we designed a global metabolomic study to compare the metabolome of two different breast cancer cell lines, MCF-7 and MDA-MB-231, in response to genistein (Gen), daidzein (Dai) and a soy seed extract (Ext). MCF-7 are low invasive, estrogen-responsive cells, while MDA-MB-231 are high invasive, estrogen non-responsive breast cancer cells. Given that soy isoflavones display dose-dependent effects, we selected, for all of our compounds, concentrations that led to a 20% inhibition of cell growth compared to control (IC_20_) and to a 20% stimulation of cell growth compared to control (SC_20_), respectively. To our knowledge, this is the first study to use specific concentrations for each test compound in order to obtain comparable effects. This subject is highly significant as isoflavones have different binding capacities and potencies [8].

Therefore, the purpose of our project was: (i) to evaluate the specific metabolomic changes induced by each test compound, in relation to the control; (ii) to identify which metabolic modulations are specific to breast cancer cell inhibition or proliferation, by comparing the metabolic profile of cells exposed to IC_20_ or SC_20_ of test compounds; and (iii) to identify which modulations are dependent on the estrogen receptors (ER), by comparing the metabolome of MCF-7 cells to the metabolome of MDA-MB-231, exposed to the same test compound, at equivalent concentrations.

## 2. Results and Discussion

### 2.1. Evaluation of Cell Viability

**Figure 1:**
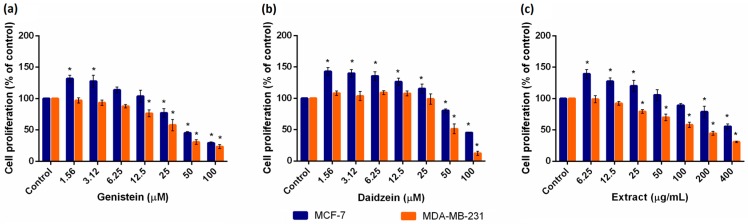
Effects of (**a**) genistein (Gen), (**b**) daidzein (Dai) and (**c**) a soy seed extract (Ext) on the proliferation of MCF-7 and MDA-MB-231 cells evaluated using the 3-(4,5-dimethylthiazol-2-yl)-2,5-diphenyltetrazolium bromide (MTT) assay. Cells were exposed to the indicated concentrations of test compounds for 72 h. Control cells were exposed only to solvent (Dimethyl sulfoxide (DMSO) 0.4%). Results were expressed as a percentage of the solvent control (set as 100%) and represented as the mean ± standard deviation (SD) of four independent experiments; * *p* < 0.001 compared to the corresponding control according to one-way analysis of variance (ANOVA) test followed by Dunnett’s multiple-comparison test.

An MTT assay was conducted in order to evaluate the metabolic activity of breast cancer cells after exposure to isoflavones. All test compounds induced a biphasic effect on estrogen-responsive MCF-7 cells, as presented in Figure 1.

At relatively low concentrations (1.56–13.06 µM for Gen, 1.56–34.28 µM for Dai and 6.25–67.61 µg/mL for Ext), isoflavones stimulated the cell growth compared to control, while higher concentrations had an inhibitory effect. It is well known that isoflavones exhibit a two-fold effect on estrogen responsive cells, stimulating the cell growth at low concentrations (0.1–10 µM for Gen) and causing inhibition at higher concentrations [9,10]. The proliferative effects of isoflavones are estrogen receptor mediated, while the inhibitory effects are considered to be estrogen independent [9]. These inhibitory effects result from different molecular mechanisms, as high isoflavone doses (usually >20 µM for Gen) have been shown to trigger apoptosis, inhibit cell proliferation and survival or elicit antioxidant and antiangiogenetic effects in breast cancer cells [11].

Notably, concentrations of Gen below 5 µM correspond to plasmatic concentrations attainable after a soy-rich diet [12]. Thus, to avoid any stimulatory growth effect, patients with ER breast cancer or those with a high breast cancer risk should pay special attention to their daily phytoestrogen intake.

For MDA-MB-231 cells, as they do not express estrogen receptors, only a dose-dependent inhibitory effect was observed. Moreover, MDA-MB-231 cells proved to be more sensitive than MCF-7 cells, since the same isoflavone concentrations caused a greater inhibitory effect in MDA-MB-231 cells compared to MCF-7 cells. Dose-response curves were plotted for each test compound (Supplementary Material, Section 1, Figure S1). Based on these curves, we selected the concentrations for the following metabolomic experiments. For MCF-7 cells, we selected the test concentrations that resulted in a 20% higher proliferation compared to control (SC_20_) and the test concentrations that inhibit cell growth by 20% compared to control (IC_20_). For MDA-MB-231 cells, only the IC_20_ concentrations were selected (Table 1).

### 2.2. Metabolite Profiling of Breast Cancer Cells Exposed to Isoflavones

MCF-7 and MDA-MB-231 cells were treated with the selected concentrations of Gen, Dai and Ext for 72 h.

For sampling and extraction, cells were directly scrapped in a 1:1 methanol:water mixture. This was reported to be the method of choice for harvesting adherent cells and extract cellular metabolites due to the good recovery and the high metabolite yielded [13]. Next, all samples were submitted to ^1^H-NMR and GC-MS analysis.

#### 2.2.1. Extracellular Metabolome

**Figure 2:**
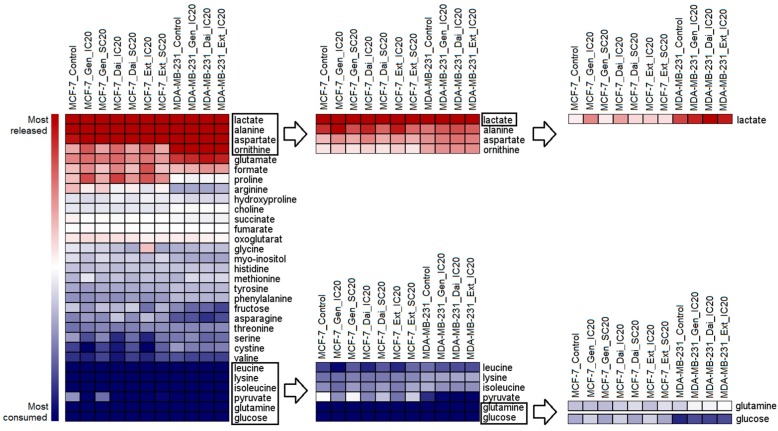
A hierarchical heat map displaying the differences between the most released (represented in red color) and the most consumed (represented in blue color) extracellular metabolites after exposure to isoflavones. The outlined metabolites are represented in the right heat maps in a more detailed view. For each metabolite, the relative concentration was normalized using Equation 1 and represented as the mean of five independent experiments. For hierarchical clustering, the optimized gene leaf order was selected, and the distance was measured using the Euclidean algorithm.

Following ^1^H-NMR analysis, 31 extracellular metabolites were identified and relatively quantified to the area of the internal standard. For each metabolite, the difference in relative concentration from conditioned to fresh medium was calculated and normalized to the yielded cell number (see Equation (1) and Supplementary Material, Table S1). Based on these, the metabolite consumption and the release rates/cell were obtained for each treatment condition. The profile of extracellular metabolites after normalization is presented in Figure 2.

In all samples, the most consumed metabolites were glucose and glutamine, the main carbon sources necessary for adenosine triphosphate (ATP) generation and biosynthetic processes. The next most consumed nutrient was pyruvate, which was added to the medium as an additional energy source. Due to the increased aerobic glycolysis specific to cancerous cells, glucose and pyruvate are largely converted to lactate. The extracellular lactate can originate also from the intensified glutaminolytic flux, which is accompanied by a high glutamate, alanine and aspartate release, as observed in all samples.

While specific metabolic alterations between estrogen-dependent and independent cell lines have been largely investigated [14,15,16], we focused our study on detecting the isoflavone-induced effects. Therefore, we related the extracellular metabolite profiles of each treated sample to the solvent control of the respective cell line (Table S1).

**Figure 3:**
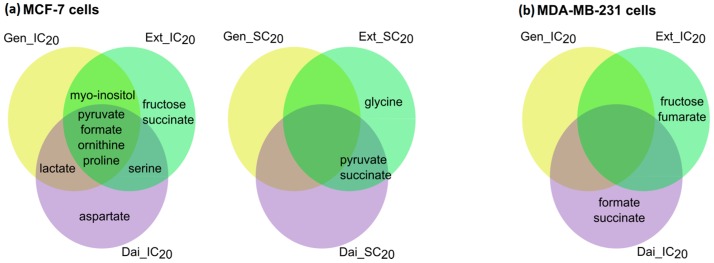
Venn-diagrams presenting the unique and shared extracellular metabolites that were significantly altered after exposing (**a**) MCF-7 cells and (**b**) MDA-MB-231 cells to Gen (yellow spot), Dai (purple spot) or Ext (green spot) at the selected test concentrations. Significantly changed metabolites compared to the control were selected based on a multiple *t*-test (α = 0.01) and a fold change (FC) threshold of 1.5 (see Table S1).

For MCF7 cells, IC_20_ of the test compounds induced greater alterations in the extracellular metabolome than SC_20_ (Figure 3). After exposure to IC_20_ of Gen, Dai or Ext, similar changes in pyruvate uptake and formate, ornithine and proline release were observed, suggesting that similar pathways were affected. The enhanced pyruvate uptake was associated with increased lactate release. A significant increase in lactate secretion was observed only when cells were exposed to IC_20_ of Gen or Dai. Most probably, ornithine, proline and serine alterations reflect intracellular modifications in the amino acid biosynthesis or degradation. Notably, after Ext treatment (IC_20_ or SC_20_), most metabolites were significantly changed.

For MDA-MB-231 cells, no significant alteration was seen following Gen treatment (IC_20_), but a decrease in glucose and glutamine consumption was observed. IC_20_ of Ext and Dai induced distinct changes that partially superposed with the exometabolome changes that occurred in MCF-7 cells after exposure to the same test concentrations. Thus, it is possible that similar molecular mechanisms trigger cell inhibition in both cell lines.

#### 2.2.2. Intracellular Metabolome

By using GC-MS, 81 intracellular metabolites were detected in MCF-7 cells, of which 79 were identified and relatively quantified. For MDA-MB-231 cells, 73 out of 74 intracellular metabolites were identified and relatively quantified (Supplementary Material, Table S2). These metabolites belong to various chemical classes, including amino acids, sugars or fatty acids, and are key intermediates in different metabolic pathways, such as glycolysis, citric acid cycle, amino acid metabolism or glycerolipid metabolism.

**Figure 4:**
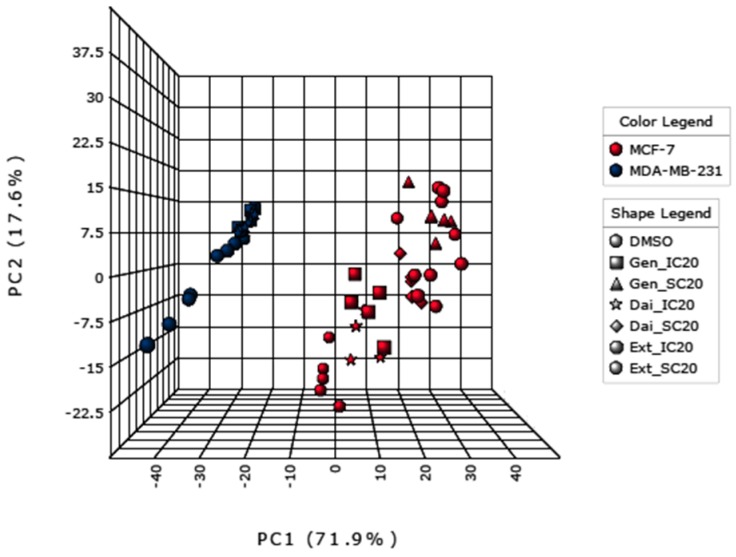
Principal component analysis (PCA) displaying the main sources of variances: PC1, 71.9% of total variance, is attributed to the cell types, while PC2, 17.6% of total variance, is due to the treatment (test substance and concentration).

To evaluate the major patterns within the data, we performed a principal component analysis (PCA) (Figure 4). The first principal component, representing 71.9% of the total variance, clearly attributes the differences to the two genotypes. The second principal component, responsible for 17.6% of the variance, reflects the differences between the treatment conditions.

Next, the intracellular metabolite profile of each treated sample was compared to the intracellular profile of the control for the respective cell line. By excluding cell type differences, specific metabolic alternations induced by Gen, Dai or Ext can be detected and evaluated.

##### Intracellular Metabolome of MCF-7 Cells

MCF-7 cells were exposed to Gen, Dai or Ext at IC_20_ and SC_20_ levels. In order to evaluate the metabolic pathways influenced by isoflavone treatment, two approaches were considered.

**Figure 5:**
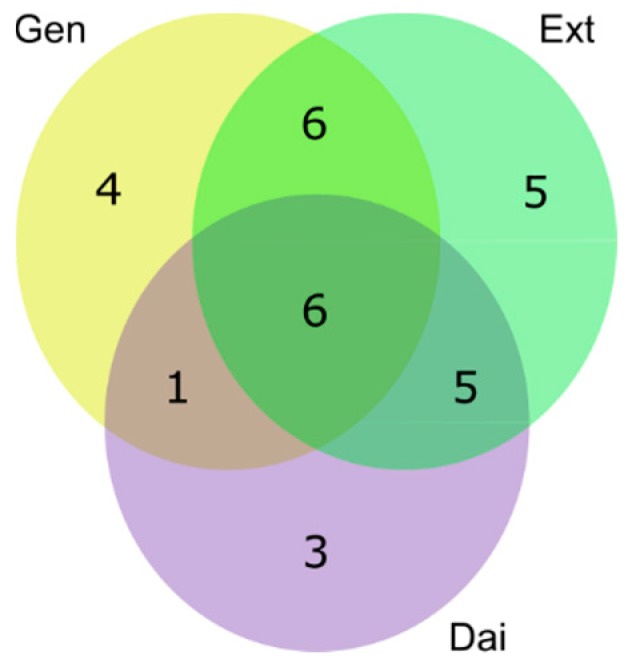
Venn-diagram presenting the number (1–6) of the unique and shared intracellular metabolites that were significantly changed after exposing MCF-7 cells to Gen (yellow spot), Dai (purple spot) and Ext (green spot). Significantly-changed metabolites were selected based on a multiple *t*-test (α = 0.01) and an FC threshold of 1.5 (see Table S2).

In the first approach, we compared the metabolic effects induced by the test compounds individually, without regard to the test concentration. By selecting all significantly-changed metabolites following Gen, Dai or Ext treatment, at both low (SC_20_) and high (IC_20_) doses, we generated specific metabolite sets for each test compound, which covered a wide range of exposure doses. Moreover, using this approach, we were able to identify the shared and unique metabolite patterns between test compounds and to predict the similar and the specific altered pathways (Figure 5).

As seen in Figure 5, most of the significantly-changed metabolites are common to all test compounds, suggesting that most of the altered metabolic pathways are common. The Ext generated most of the significantly-changed metabolites, comprising the metabolites induced by Gen and/or Dai. Therefore, the metabolic changes induced by the Ext are, at least partly, due to the Gen and Dai content.

Of the six common altered metabolites, 3-phosphoglycerate and phosphoenolpyruvate are involved in glycolysis; aspartate and serine are involved in the amino acids metabolism, while glycerol 3-phosphate and myo-inositol are part of the glycerolipid metabolism and inositol metabolism, respectively. Beside these common metabolites, the Ext significantly influenced the concentration of five other metabolites, namely glucose 6-phosphate, d-ribose 5-phosphate, 4-hydroxyproline, pantothenate and asparagine. Thus, the Ext can exert additional mechanisms of action, due to the synergic effect of Gen and Dai or to other active compounds.

As most of the significantly-changed metabolites were common to all test compounds, we focused, in our second approach, on discriminating between the metabolic alterations induced at IC_20_ and SC_20_, respectively, without regard to the test compound. By selecting the significantly-changed metabolites following IC_20_ and SC_20_ treatment, irrespective of the test compound, we highlighted the metabolic features specific to the inhibitory and stimulatory growth effects. The comparison is reliable, as we used equivalent inhibitory and stimulatory doses for each compound (the IC_20_ and SC_20_, respectively).

**Figure 6:**
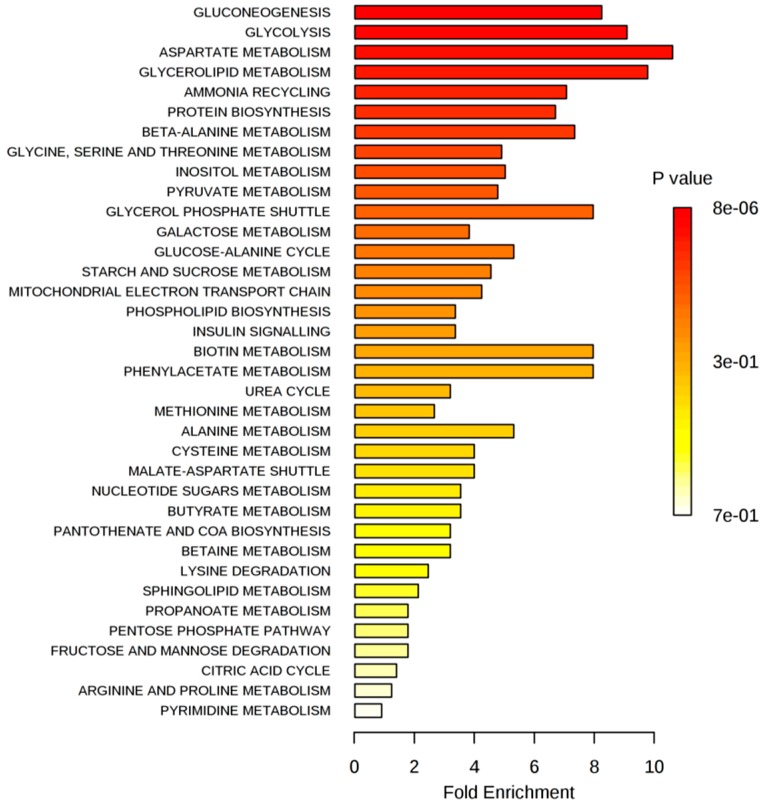
Summary plots for over-representation analysis (ORA) of significantly-changed metabolites after exposing MCF-7 cells to the IC_20_ of test compounds. Molecular pathways were ranked according to the probability (*p*-value) of finding a particular number of significantly-changed metabolites in the compound list of a certain pathway. ORA was implemented using the hypergeometric tests.

The list of significantly changed metabolites that resulted after exposing MCF-7 cells to IC_20_ of test compounds were selected based on the multiple *t*-test (α = 0.01) and an FC threshold of 1.5 (Table S2). Metabolic pathway analysis was conducted by submitting this list to over-representation analysis (ORA), provided by MetaboAnalyst 3.0 [17]. ORA is a type of metabolite set enrichment analysis (MSEA), which evaluates whether a subset of metabolites is associated with a particular pathway more than just by chance. The ORA summary plots are presented in Figure 6 (the complete MSEA reports are available in the Supplementary Material, Section 2).

As presented in Figure 6, the most associated metabolic pathways after exposing MCF-7 cells to the IC_20_ of test compounds were gluconeogenesis/glycolysis, aspartate and glycerolipid metabolism.

**Figure 7:**
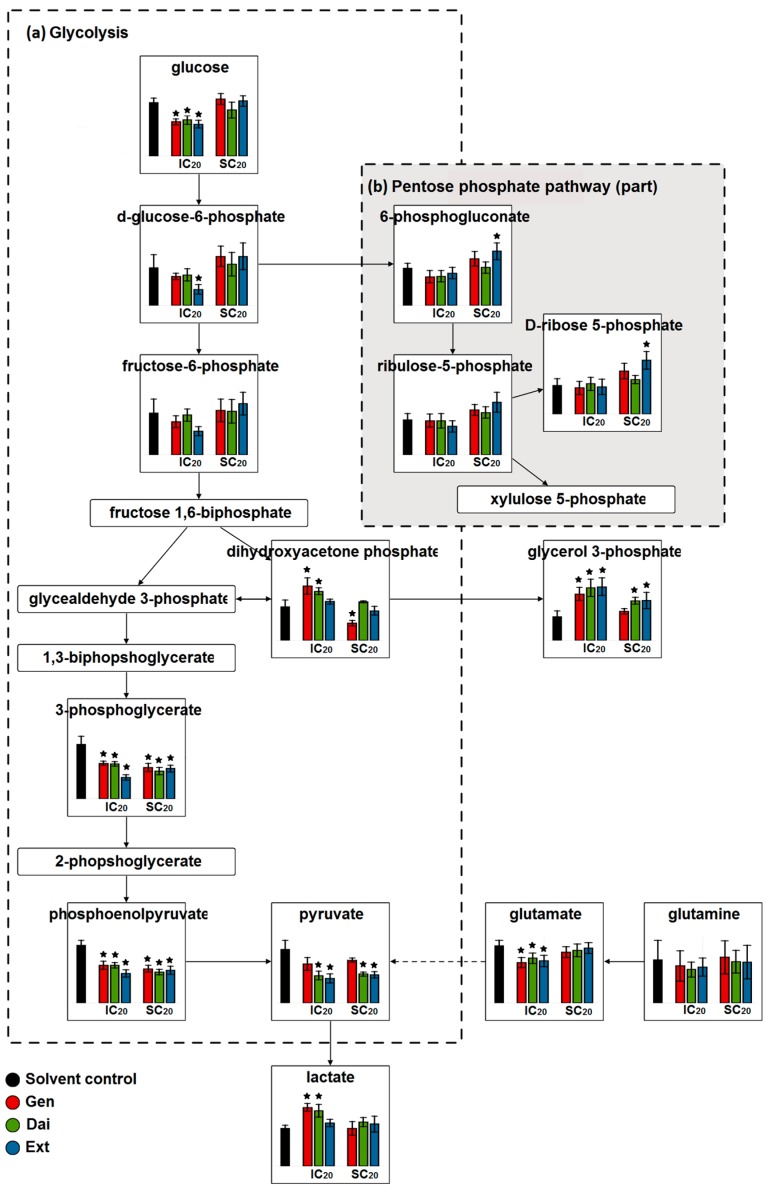
The relative concentrations of the metabolites involved in the (**a**) glycolytic pathway and (**b**) pentose phosphate pathways (part), after exposing MCF-7 cells to the IC_20_ and SC_20_ of the test compounds. Data are expressed as the mean value ± SD of five independent experiments; * indicate statistically-significant differences (*p* < 0.01, Student’s *t*-test) between the control and treated samples.

Despite the apparent inefficiency in terms of ATP production, glycolysis represents the energy generator on which breast cancer cells rely. Glucose degradation supplies cells with intermediates required for biosynthetic pathways, such as ribose sugars for nucleotides or 3-phosphoglycerate for membrane lipids. Therefore, any imbalance in the glycolytic flux can either exhaust cells of essential intermediates contributing to cell death or, conversely, can sustain the biosynthetic needs of high proliferating cells. To get a deeper understanding of how different isoflavone doses interfere in the glycolytic pathway, we compared the relative concentrations of glycolysis metabolites after exposing MCF-7 cells to inhibitory and stimulatory doses of isoflavones (Figure 7).

Inhibitory concentrations of test compounds decreased the glucose and glutamine uptake, as confirmed by our extracellular data. While intracellular glucose was significantly reduced (with approximately 30% compared to control), glutamine uptake was only moderately inhibited. Stimulatory concentrations of test compounds enhanced glucose uptake, but had no significant effect on the glutamine uptake.

Glucose transport over the cell membrane represents a rate-limiting step for glucose metabolism and is controlled mainly by glucose transporters (GLUTs), of which GLUT1 is the predominant isoform. It was proven that Gen (10–100 µM), similar to other dietary polyphenols, can decrease GLUT1-mediated glucose uptake in breast [18,19] and prostate [20] cancer cells. Apparently, Gen can act either directly, by decreasing the affinity of glucose for the external binding site of GLUT1 [21], or by modulating the signaling pathways responsible for GLUT1 regulation, such as the phosphatidylinositol 3-kinase Akt (PI3K/Akt) pathway [22] or the hypoxia-inducible factor 1 (HIF-1) [23].

Inhibition of glucose usage following exposure to the IC_20_ of test compounds was expected to correlate with a reduction of the intracellular lactate. However, the level of intracellular lactate increased. Similar elevated levels were observed for the extracellular lactate, as seen in the ^1^H-NMR data.

In addition, the IC_20_ of test compounds favored dihydroxyacetone phosphate (DHAP) accumulation and elevated glycerol 3-phosphate levels. As the balance is tipped towards glycerol 3-phosphate formation, less DHAP is isomerized to glyceraldehyde 3-phosphate to generate ATP in the pay-off phase of glycolysis. On the other hand, glycerol 3-phosphate is also the main precursor for glycerophospholipids, required for membrane phospholipid synthesis.

Several studies have shown that Gen interferes in the metabolism of lipids, especially in the sphingolipid metabolism. Nanomolar concentrations of Gen promoted sphingolipid metabolism by inducing the expression of acid ceramidase, a key gene in controlling ceramide/sphingosine/sphingosine-1-phosphate balance [24]. Exposure of MCF-7 cells to higher doses of Gen (>10 µM) or a flax extract of *Linum usitatissimum* (>1 µg/mL) increased the expression of sphingosine-1-phospate lyase, enhancing the degradation of sphingosine-1-phospate [7]. Therefore, isoflavones, depending on their concentration, can entail modulations also in the lipidic balance. When MCF-7 cells were exposed to the SC_20_ of test compounds, the glucose uptake was slightly stimulated. As the relative concentrations of 3-phosphoglycerate decreased significantly, we sought for alternative pathways where the upstream glucose might have been consumed. We observed an increased level of 6-phosphogluconate and ribose 5-phosphate (Figure 7b), supporting the idea that glucose 6-phosphate is partially redirected towards the pentose phosphate pathway (PPP). By upholding the oxidative PPP branch, ribose-5-phosphate and reduced nicotinamide adenine dinucleotide phosphate (NADPH) are generated, both essential for de novo nucleotide biosynthesis.

Similar modulations were described after MCF-7 cells were exposed to estradiol [25]. Estradiol stimulation significantly increased the transcript levels of glucose-6-phosphate dehydrogenase (G6PD), promoting glucose metabolism through PPP [25,26]. As isoflavones are estrogen-like compounds, it is likely that their growth-promoting effects induce the same metabolic changes, upregulating G6PD and increasing the flux through the PPP.

##### Intracellular Metabolome of MDA-MB-231

**Figure 8:**
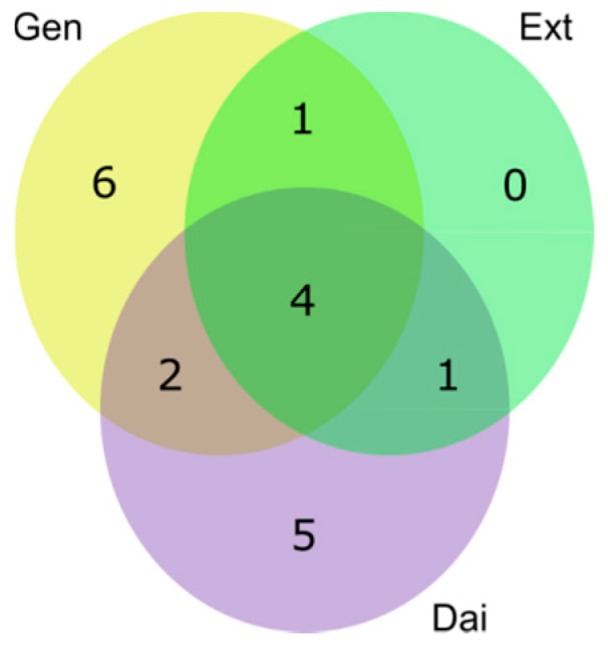
A Venn-diagram presenting the number (1–6) of the unique and shared intracellular metabolites that were significantly changed after exposing MDA-MB-231 cells to Gen (yellow spot), Dai (purple spot) and Ext (green spot). Significantly-changed metabolites were selected based on a multiple *t*-test (α = 0.01) and an FC threshold of 1.5 (see Table S2).

MDA-MB-231 cells were exposed to IC_20_ of Gen, Dai and Ext. To evaluate the metabolic changes that occurred after isoflavone treatment, first, we sought for particular metabolic signatures induced by each test compound, individually. As presented in Figure 8, exposure to Gen or Dai induced the most significant changes in the metabolome of MDA-MB-231 cells. Notably, the Ext did not induce characteristic alterations, the significantly-changed metabolites being common to Gen or/and Dai.

The significantly-changed metabolites common to all test compounds were glutamine, fructose, beta-alanine and putrescine. Gen caused particular changes in the concentration of 2-oxoglutarate, 6-phosphogluconate, glucose, glycerol 3-phosphate, leucine and valine, while Dai induced specific changes in alanine, cystathionine, hypotaurine, phosphoenolpyruvate and succinate levels. As most altered metabolites were involved in the protein biosynthesis or glutamate metabolism, it is assumed that these pathways represent the main metabolic targets of isoflavones in MDA-MB-231 cells.

**Figure 9:**
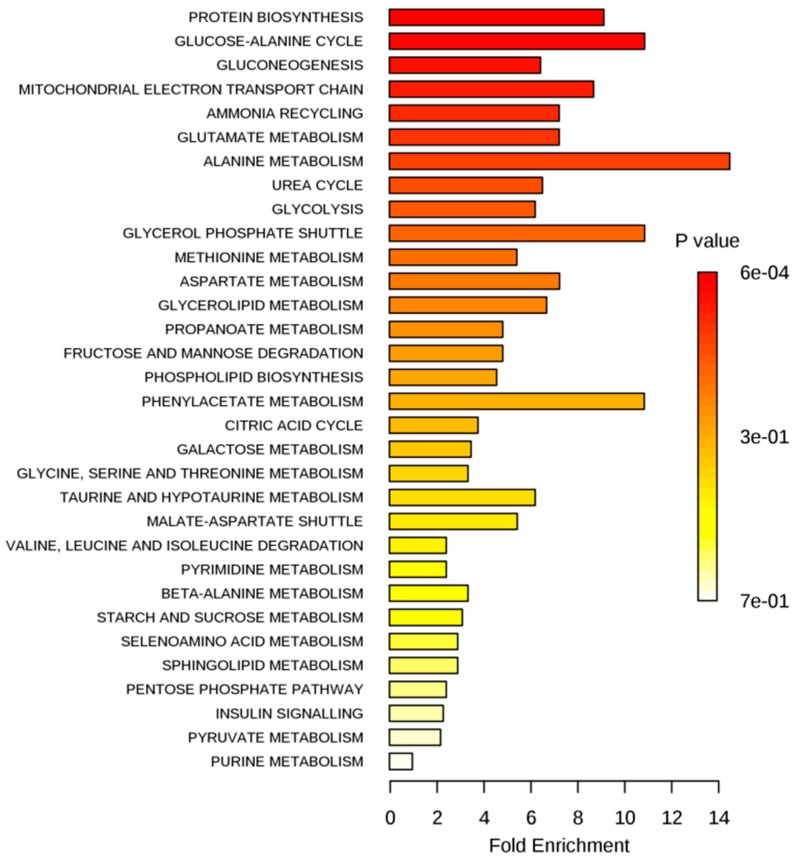
Summary plots for ORA of significantly-changed metabolites after exposing MDA-MB-231 cells to Gen, Dai or Ext. Molecular pathways are ranked according to the probability (*p*-value) of finding a particular number of significantly-changed metabolites in the compound list of a certain pathway. ORA was implemented using the hypergeometric tests.

Our observations were confirmed by the metabolic pathway analysis using ORA. The significantly-changed metabolites, selected based on the multiple *t*-test (α = 0.01) and an FC threshold of 1.5 (Table S2), generated the ORA summary plots presented in Figure 9 (the complete MSEA reports are available in the Supplementary Material, Section 3).

**Figure 10:**
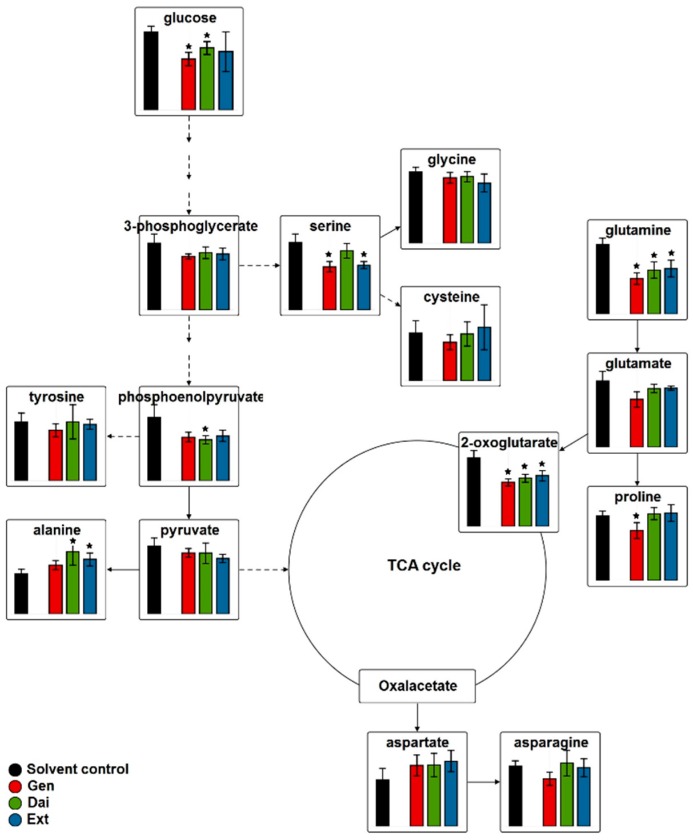
The relative concentrations of the amino acids after exposing MDA-MB-231 cells to the IC_20_ of test compounds. Data are expressed as the mean value ± SD of five independent experiments; * indicate statistically-significant differences (*p* < 0.01, Student’s *t*-test) between the control and treated samples. TCA: tricarboxylic acid cycle.

To obtain an overview of the amino acids’ alterations upon isoflavone treatment, the data were integrated into a metabolic pathway map (Figure 10).

After isoflavone treatment, the concentration of asparagine, cysteine, glutamate, glutamine, glycine, proline, serine and tyrosine generally decreased, while the concentration of alanine and aspartate increased. Although the biosynthesis of these non-essential amino acids is different, their carbon skeletons are derived from the intermediates of common pathways, namely glycolysis, the citric acid cycle or glutaminolysis.

In the glycolytic pathway, the IC_20_ of test compounds triggered comparable alterations as for MCF-7 cells. Principally, the concentration of intracellular glucose decreased compared to the control, especially following Gen treatment. Impairment of glucose uptake resulted in fewer precursors and less ATP necessary for proliferation and cell growth. The control of glucose transport across the plasma membrane is carried out by the same GLUTs, highly expressed in MDA-MB-231 cells.

Two flavonoids, quercetin and epigallocatechin gallate, have been shown to inhibit GLUTs-mediated uptake of glucose in breast cancer cells, independently of estrogen signaling [27]. Isoflavones seem to act similarly, as GLUTs inhibitors, limiting glucose uptake in both ER-positive and ER-negative breast cancer cells.

Still, the glucose uptake was more strongly inhibited in MCF-7 cells, where the glucose level decreased by 35%, 32% and 40% following Gen, Dai and Ext treatment, respectively, compared to MDA-MB-231 cells, where the same test compounds reduced the glucose uptake by only 33%, 20% and 24%, respectively.

Moreover, we observed a significant decrease in glutamine uptake, which was confirmed by our extracellular data.

Glutamine, as glucose, represents a major fuel for cancer cells, providing metabolic intermediates to be channeled into the citric acid cycle and precursors for the biosynthesis of several amino acids, nucleic acids and glutathione. Furthermore, triple negative breast cancer cells exhibit “glutamine addiction”, being dependent on glutamine to support cell growth and activation of the target of rapamycin kinase [28]. Therefore, MDA-MB-231 cells strongly rely on glutamine uptake, any decrease in glutamine level impairing the cell growth.

Glutamine transport across cell membrane is controlled by alanine, serine, a cysteine-preferring transporter 2 (ASCT2) and a cell surface solute-carrying transporter. Recently, it was shown that triple negative breast cancer cells require ASCT2-mediated uptake of glutamine to sustain mTORC1 signaling, cell growth and cell cycle progression [29].

To our knowledge, similar observations in glutamine-restricted uptake were described only after curcumin treatment (50 µM for 48 h) of colorectal cancer stem cells. Curcumin induced apoptosis and cell death, which was metabolically reflected into a defective glutamine transport into the cells [30]. Given the limited number of natural inhibitors of ASCT2, the decreased glutamine concentration observed in our study is, most likely, due to the modulation of more upstream regulators, rather than a direct inhibition of ASCT2 membrane transporters.

ASCT2 expression is regulated by a number of cancer genes, including c-Myc, ATF4 or N-Myc [29,31]. There is consistent evidence in the literature sustaining that Gen can modulate c-Myc expression [32,33], inactivate the PI3K/Akt/mTOR pathway [34] or modulate the Ras/Raf-1/ERK1/2 pathway [35]. However, further studies are required to investigate the specific impact of isoflavones on ASCT2 transporters and glutamine metabolism.

Notably, the derived glutamate does not seem to participate in replenishing the citric acid cycle (anaplerosis) via 2-oxaloacetate, since most glutamate is exported, as indicated by our ^1^H-NMR data.

In contrast to glucose, isoflavones significantly inhibited the glutamine uptake only in MDA-MB-231 cells, suggesting that the mechanisms occur only in the absence of ER.

## 3. Materials and Methods

All chemicals and standards were purchased from Sigma-Aldrich (Taufkirchen, Germany), unless otherwise stated.

The soy seed extract was received from Hunan Goldliloo Pharmaceutical Co., Ltd., Changsha, China, together with a certificate of analysis. The certificate specifies that the extract has suitable properties to be added into various health products, including food supplements. According to this certificate, the Ext contains 26.87% daidzin, 10.95% glycitin, 3.55% genistin, 1.50% Dai, 0.12% glycitein and 0.02% Gen. The extract was analyzed before and after acidic hydrolysis, and this distribution was confirmed by a validated HPLC-UV method [36].

Stock solutions of standard Gen (>98% purity), Dai (>98% purity) or Ext were prepared in dimethyl sulfoxide (DMSO) and stored at −20 °C. All chemicals were of analytical grade.

### 3.1. Cell Culture and Culture Conditions

The estrogen-sensitive human breast adenocarcinoma cell line MCF-7 and the estrogen-independent adenocarcinoma cell line MDA-MB-231 were obtained from CLS Cell Lines Service (Eppelheim, Germany). All cells were routinely cultured in RPMI 1640 medium supplemented with 10% heat-inactivated fetal bovine serum, 1 mM sodium pyruvate, 0.1 mM non-essential amino acids and 1% penicillin-streptomycin. Cells were incubated at 37 °C in a humidified 5% CO_2_ atmosphere. Both cell lines were maintained as monolayers in 75 cm^2^ plastic flasks (TPP, Trasadingen, Switzerland). After reaching confluence, the cells were washed twice with 3 mL phosphate buffer saline (1× PBS) and incubated with 3 mL 0.05% trypsin/0.02% EDTA for 1 min. The trypsin treatment was halted by adding complete media, and the cells were centrifuged (230× *g*, 3 min) and resuspended in complete medium. The cell number and viability were determined using the Casy Model TT (Roche Life Sciences, Indianapolis, IN, USA).

### 3.2. MTT Assay

Prior to metabolomic experiments, the cytotoxic potential of the test compounds was evaluated using the 3-(4,5-dimethylthiazol-2-yl)-2,5-diphenyltetrazolium bromide (MTT) assay.

MCF-7 and MDA-MB-231 cells were plated in 96-well plates (TPP, Trasadingen, Switzerland) at a density of 1500 cells/well in complete growth medium. After 24 h of incubation, the cells were washed with Hank’s Balanced Salt Solution (HBSS) and incubated in medium containing serial dilutions of Gen, or Dai (1.56–100 µM), or Ext (6.25–400 µg/mL). A blank control (medium only), a solvent control (DMSO 0.4%) and a positive control (Etoposide 5 µM for MCF-7 cells and Etoposide 1.9 µM for MDA-MB-231 cells) were included in every plate. Each treatment had eight replicates per plate. After 72 h of incubation, cells were washed again with HBSS and incubated for 3 more hours with medium containing 0.5 mg/mL MTT. The resultant formazan was dissolved in 200 µL DMSO, and absorbance was measured at 550 nm (Varioskan Flash Multimode Reader, Thermo Scientific, Waltham, MA, USA). Each experiment was repeated four times. Details about the assessment of cell viability are available in the Supplementary Material, Section 1.

### 3.3. Sample Preparation for Metabolomics

#### 3.3.1. Cell Treatment

For each cell line, cells were identically seeded in two cell culture dishes and exposed to the same treatment conditions. More precisely, 2.4 × 10^6^ MCF-7 cells or 1.2 × 10^6^ MDA-MB-231 cells were seeded in 150-mm cell culture dishes (Sarstedt, Nümbrecht, Germany) in 15 mL complete growth medium. Dishes were then incubated for 24 h to allow cell attachment. After incubation, the medium was replaced with 28 mL fresh medium containing the selected concentrations of Gen, Dai, Ext or DMSO as the solvent control. In all cases, the final concentration of DMSO in medium did not exceed 0.01%. After 72 h of incubation, one plate was used for subsequent sampling experiments and one served for counting the cells using Casy Model TT. All experiments were performed in quintuplicate.

Due to prolonged exposure, preliminary studies were carried out in order to ensure that at the end of the experiment, the cells were still viable and capable of proliferating. For this, we assessed the cell confluence every 24 h by visually examining the cells under the microscope. Furthermore, we ensured that the medium still contained enough nutrients after 72 h by measuring the concentration of remaining glucose, glutamine and essential amino acids using ^1^H-NMR (Bruker Biospin, Karlsruhe, Germany).

#### 3.3.2. Sampling and Metabolism Quenching

For medium sampling, 1.5 mL cell medium were filtered using a 0.2-µm pore size filter (Sarstedt, Nümbrecht, Germany) and immediately frozen. The filtrates, representing the extracellular metabolite samples, were stored at −20 °C until measurement.

The cell sampling, quenching and extraction steps followed the procedures described by Gierok et al. [37], with minor modifications. Briefly, the medium was discarded, and the cell monolayer was washed four times with a total of 50 mL of ice-cold NaCl (135 mM). Next, 400 µL internal standard containing 40 nmol ribitol were added directly over the cells. Subsequently, 10 mL of ice-cold methanol were added, and cells were rapidly scraped and transferred into a 50-mL Falcon tube. The plate was washed again, this time with 10 mL of ice-cold distilled water, and the suspension was transferred into the same Falcon tube. Immediately afterwards, the samples were frozen in liquid nitrogen and stored at −80 °C.

#### 3.3.3. Metabolite Extraction and Lyophilization

For extraction, samples were allowed to thaw on ice. Next, 2 mL chloroform were added, and samples were vortexed for 1 min to favor extraction. The extraction samples were incubated in ice for 10 min and then centrifuged for 5 min, at 4000 rpm, 4 °C. Following centrifugation, the aqueous-methanolic phase was separated and transferred into a different Falcon tube, frozen in liquid nitrogen and stored at −80 °C. Prior to analysis, samples were lyophilized at −52 °C and 0.54 mbar (Christ Alpha 1–4 LD Freeze Dryer, Osterode am Harz, Germany).

### 3.4. ^1^H-NMR Analysis

For the analysis of extracellular metabolites, ^1^H-nuclear magnetic resonance (^1^H-NMR) analysis was performed, using the method described before [38]. Briefly, 400 µL medium filtrate were mixed with 200 µL NMR buffer solution containing 1 mM of 3-(trimethylsilyl)propionic-2,2,3,3-*d*_4_ acid sodium salt as an internal standard. Samples were analyzed using a Bruker Avance II-600NMR spectrometer (Bruker Biospin, Karlsruhe, Germany), operating at a proton frequency of 600.27 MHz. All NMR spectra were recorded using TOPSPIN 3.2 software (Bruker Biospin), and metabolite signals were referenced according to the internal standard.

Data analysis was carried out using AMIX v3.9.12 software (Bruker Biospin), as described [38]. The relative concentration of each metabolite was normalized using the following formula, adapted after Jain et al. [39]:

(1) $$\frac{{\left[C\right]}_{72h}-{\left[C\right]}_{t0}}{N_{72h}}$$

[*C*]_72h_, the relative concentration after exposure to the test compound or the control for 72 h; [*C*]_t0_, the relative concentration in the initial medium (*t*_0_); *N*_72h_, the cell number after 72 h exposure to the test compound or the control.

### 3.5. GC-MS Analysis

For the analysis of intracellular metabolites, the lyophilized samples were derivatized with methoxyamine and *N*-methyl-*N*-(trimethylsilyl) trifluoroacetamide (Chromatographie-Service GmbH) as described [40]. After derivatization, samples were analyzed using an Agilent 6890N GC System coupled with a 5973 Network Mass Selective Detector (Agilent Technologies, Santa Clara, CA, USA). The GC-MS analysis was carried out using the parameters previously established [40], except for the oven program setup, which was slightly modified: the holding time after the last temperature ramp (20 °C/min up to 330 °C) was increased from 3 to 8 min, in order to ensure the complete elution of compounds.

Identification and relative quantification of intracellular metabolites were performed using ChromaTOF software (LECO Corporation, St. Joseph, MI, USA). A metabolite was considered correctly identified if the peak mass spectra showed a match factor ≥750 to the corresponding library entry and had a retention time deviation within 2 s. For relative quantification, the areas of the identified peaks were divided according to the area of the internal standard. This ratio, representing the relative amount of each metabolite, was normalized to the cell number.

### 3.6. Statistics and Visualization

Statistical analysis was executed using Prism (v 6.01, GraphPad Software, La Jolla, CA, USA) and MetaboAnalysit 3.0 [17]. The charts were created using Microsoft Excel 2013 and Prism (v 6.01, GraphPad Software). Principal component analysis (PCA) and ORA were executed using MetaboAnalyst 3.0. Hierarchical clustered heatmaps were created with MeV (v 4.9, Boston, MA, USA), using Euclidean’s correlation coefficient for distance measurement. For pathway mapping, we used VANTED (v 2.5.3, Seeland, Germany).

## 4. Conclusions

This study presents, for the first time, the global metabolome profiles of MCF-7 estrogen-responsive and MDA-MB-231 estrogen non-responsive breast cancer cells exposed to various doses of genistein, daidzein or a soy seed extract.

Extracellular and intracellular data confirmed that most of the altered metabolites were common to all test compounds, suggesting that the metabolic pathways affected by isoflavones were communal and the metabolic effects of the Ext were mostly due to the Gen and Dai content. However, inhibitory doses of Ext induced alterations to a larger number of metabolites in MCF-7 cells, and therefore, other inhibitory mechanisms might be involved, as well.

Exposure of MCF-7 cells to low isoflavone doses (SC_20_) induced estrogen-like effects, stimulating the cell proliferation. In order to sustain the increased biosynthetic needs, isoflavones appeared to redirect a part of glucose-6-phosphate towards PPP, ensuring cells with more ribose sugars and NADPH. Exposing MCF-7 and MDA-MB-231 cells to high isoflavones doses (IC_20_) hindered cell proliferation. In both cell lines, isoflavone exposure led to a deficient glucose uptake, depriving cells of their main energy source. The glucose influx was restricted more in MCF-7 cells than in MDA-MB-231 cells. However, the glutamine uptake was significantly inhibited only in MDA-MB-231 cells. As MDA-MB-231 cells are considered “glutamine addicted”, an impaired glutamine uptake, correlated with a defective glucose import, led to alterations of protein biosynthesis.

Collectively, these data indicate that isoflavones elicited dose-dependent effects, triggering either breast cancer cell proliferation or cell death. These cellular effects were strongly reflected in the cell metabolome, isoflavones modulating mainly the glucose uptake. Using the correct dose and exploiting the multiple molecular mechanisms of isoflavones can represent a powerful weapon in breast cancer chemoprevention.

## Figures and Tables

**Table 1:** The concentrations of genistein (Gen), daidzein (Dai) and a soy seed extract (Ext) selected for the metabolomic experiments.

Test Compound	MCF-7		MDA-MB-231
	SC_20_	IC_20_	IC_20_
Gen (µM)	5.62	22.44	11.04
Dai (µM)	19.01	52.24	36.39
Ext (µg/mL)	22.59	166.34	26.36

SC_20_, IC_20_: concentrations of Gen, Dai and Ext that induced a 20% growth stimulation or inhibition, respectively, compared to the control.

## Supplementary Materials

Supplementary materials can be found at www.mdpi.com/1422-0067/17/9/1443/s1.

## Abbreviations

^1^H-RMN	Proton nuclear magnetic resonance
AFT4	Activating transcription factor 4
ASCT2/SLC1A5	Alanine, serine, cysteine-preferring transporter 2
ANOVA	Analysis of variance
ATP	Adenosine triphosphate
Dai	Daidzein
DHAP	Dihydroxyacetone phosphate
DMSO	Dimethyl sulfoxide
Ext	Soy seed extract
FC	Fold change
G6PD	Glucose-6-phosphate dehydrogenase
GC-MS	Gas chromatography–mass spectrometry
Gen	Genistein
GLUTs	Glucose transporters
HBSS	Hank’s Balanced Salt Solution
HIF-1	Hypoxia-inducible factor 1
IC_20_	The concentration that inhibited the cell growth by 20% compared to the control
MSEA	Metabolite set enrichment analysis
MTT	3-(4,5-Dimethylthiazol-2-yl)-2,5-diphenyltetrazolium bromide
NADPH	Nicotinamide adenine dinucleotide phosphate
ORA	Over-representation analysis
PCA	Principal component analysis
PI3K/Akt/mTOR	Phosphatidylinositol 3-kinase/Akt/mammalian target of rapamycin
PPP	Pentose phosphate pathway
SC_20_	The concentration that stimulated the cell growth by 20% compared to the control
TCA cycle	Tricarboxylic acid cycle
