# Supplementary material for: The Impact of Soy Isoflavones on MCF-7 and MDA-MB-231 Breast Cancer Cells Using a Global Metabolomic Approach
Source: Int J Mol Sci. 2016 Aug 31;17(9):1443. doi: 10.3390/ijms17091443 (PMC5037722; doi:10.3390/ijms17091443)
Supplement: Supplementary file 1 [file ijms-17-01443-s001.pdf]

Supplementary Materials: The Impact of Soy Isoflavones on MCF-7 and MDA-MB-231 Breast Cancer Cells Using a Global Metabolomic Approach

Alina Uifălean, Stefanie Schneider, Philipp Gierok, Corina Ionescu, Cristina Adela Iuga and Michael Lalk

Section 1. Assessment of Cell Proliferation Using MTT Test

The basic toxic potential of the tested isoflavones was evaluated using the 3-(4,5-dimethylthiazol-2-yl)-2,5-diphenyltetrazolium bromide (MTT) assay.

The percentage of cell proliferation was calculated using the following formula:

$$\text{Cell proliferation (\%)} = \frac{(A_{550\text{test substance}} - A_{550\text{blank}})}{(A_{550\text{solvent Control}} - A_{550\text{blank}})} \times 100 \quad (1)$$

Sigmoid dose-response curves were plotted for each test compound.

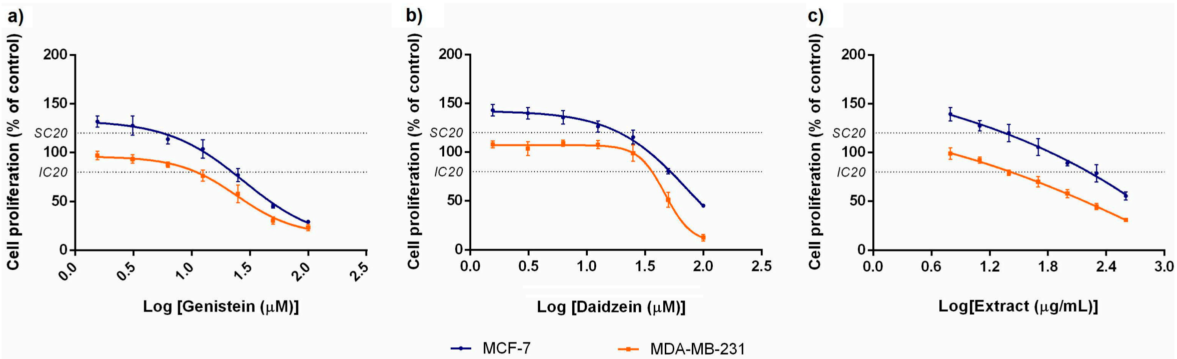

Figure S1. The dose-response curves for (**a**) genistein; (**b**) daidzein; and (**c**) soy seed extract.

Based on these curves, the SC_{20} and IC_{20} (concentrations that stimulated or inhibited, respectively, the cell proliferation by 20% compared to control) were calculated.

Section 2. MSEA Reports after Exposing MCF-7 Cells to IC_{20} of Test Compounds

Metabolomic Data Analysis with MetaboAnalyst 3.0

User ID: guest1347750194744784649

1 February 2016

1. Background

MESA or Metabolite Set Enrichment Analysis is a way to identify biologically meaningful patterns that are significantly enriched in quantitative metabolomic data. In conventional approaches, metabolites are evaluated individually for their significance under conditions of study. Those compounds that have passed certain sigificance level are then combined to see if any meaningful patterns can be discerned. In contrast, MSEA directly investigates if a set of functionally related metabolites without the need to preselect compounds based on some arbituary cut-off threshold. It has the potentail to identify subtle but consistent changes among a group of related compounds, which may go undetected with the conventional approaches.

Essentially, MSEA is a metabolomic version of the popular GSEA (Gene Set Enrichment Analysis) software with its own collection of metabolite set libraries as well as an implementation of user-friendly web-interfaces. GSEA is widely used in genomics data analysis and has proven to be a powerful alternative to conventional approaches. For more information, please refer to the original paper by Subramanian A, and a nice review paper by Nam D., Kim S.Y.

2. MSEA Overview

Metabolite set enrichment analysis consists of four steps—data input, data processing, data analysis, and results download. Different analysis procedures are performed based on different input types. In addition, users can also browse and search the metabolite set libraries as well as upload their self-defined metabolite sets for enrichment analysis. Users can also perform metabolite name mapping between a variety of compound names, synonyms, and major database identifiers.

3. Data Input

There are three enrichment analysis algorithms offered by MSEA. Accordingly, three different types of data inputs are required by these three approaches:

- A list of important compound names—entered as a one column data (Over Representation Analysis (ORA));
- A single measured biofluid (urine, blood, CSF) sample—entered as tab separated two-column data with the first column for compound name, and the second for concentration values (Single Sample Profiling (SSP));
- A compound concentration table—entered as a comma separated (.csv) file with the each sample per row and each metabolite concentration per column. The first column is sample names and the second column for sample phenotype labels (Quantitative Enrichment Analysis (QEA)).

You selected Over Representation Analysis (ORA) which requires a list of compound names as input.

4. Data Process

The first step is to standardize the compound labels. It is an essential step since the compound labels will be subsequently compared with compounds contained in the metabolite set library. MSEA has a built-in tool to convert between compound common names, synonyms, identifiers used in HMDB ID, PubChem, ChEBI, BiGG, METLIN, KEGG, or Reactome. Table 1 shows the conversion results. Note: 1 indicates exact match, 2 indicates approximate match, and 0 indicates no match. A text file contain the result can be found the downloaded file name map.csv.

Table 1. Result from Compound Name Mapping.

Number	Query	Match	HMDB	PubChem	KEGG	Comment
1	3-Hydroxybutyrate	3-Hydroxybutyric acid	HMDB00357	441	C01089	1
2	3-Phosphoglycerate	3-Phosphoglyceric acid	HMDB00807	724	C00597	1
3	4-Hydroxyproline	4-Hydroxyproline	HMDB00725	5810	C01157	1
4	Alanine	Alanine	METPA0179		C01401	1
5	Asparagine	L-Asparagine	HMDB00168	6267	C00152	1
6	Aspartate	L-Aspartic acid	HMDB00191	5960	C00049	1
7	β-Alanine	Beta-Alanine	HMDB00056	239	C00099	1
8	Butyrate	Butyric acid	HMDB00039	264	C00246	1
9	Dihydroxyacetone Phosphate	Dihydroxyacetone phosphate	HMDB01473	668	C00111	1
10	Glucose	D-Glucose	HMDB00122	5793	C00031	1
11	Gluconate	Gluconic acid	HMDB00625	10690	C00257	1
12	Glucose-6-phosphate	Glucose 6-phosphate	HMDB01401	5958	C00092	1
13	Glucuronate	D-Glucuronic acid	HMDB00127	444791	C00191	1
14	Glycerate	Glyceric acid	HMDB00139	439194	C00258	1
15	Glycerol 3-phosphate	Glycerol 3-phosphate	HMDB00126	439162	C00093	1
16	Lactate	L-Lactic acid	HMDB00190	107689	C00186	1
17	Lysine	L-Lysine	HMDB00182	5962	C00047	1
18	Methionine	L-Methionine	HMDB00696	6137	C00073	1
19	Myo-inositol	Myoinositol	HMDB00211		C00137	1
20	*N*-Acetylasparate	*N*-Acetyl-L-aspartic acid	HMDB01409	65063	C00365	1
21	Pantothenate	Pantothenic acid	HMDB00210	988	C00864	1
22	Phosphoenolpyruvate	Phosphoenolpyruvic acid	HMDB00263	1005	C00074	1

Table 1. *Cont.*

Number	Query	Match	HMDB	PubChem	KEGG	Comment
23	Pyruvate	Pyruvic acid	HMDB00243	1060	C00022	1
24	Serine	L-Serine	HMDB00187	5951	C00065	1
25	Tagatose	D-Tagatose	HMDB03418	92092	C00795	1
26	Threonate	Threonic acid	HMDB00943	151152	C01620	1

The second step is to check concentration values. For SSP analysis, the concentration must be measured in umol for blood and CSF samples. The urinary concentrations must be first converted to umol/mmol creatinine in order to compare with reported concentrations in literature. No missing or negative values are allowed in SSP analysis. The concentration data for QEA analysis is more flexible. Users can upload either the original concentration data or normalized data. Missing or negative values are allowed (coded as NA) for QEA. Please note, MSEA does not perform data normalization. If normalization is important, you should first normalize your data before upload. You can use our companion website MetaboAnalyst www.metaboanalyst.ca for a variety of data processing and normalization methods.

5. Selection of Metabolite Set Library

Before proceeding to enrichment analysis, a metabolite set library has to be chosen. There are seven built-in libraries offered by MSEA:

- Metabolic pathway associated metabolite sets (currently contains 88 entries);
- Disease associated metabolite sets (reported in blood) (currently contains 416 entries);
- Disease associated metabolite sets (reported in urine) (currently contains 346 entries);
- Disease associated metabolite sets (reported in CSF) (currently contains 124 entries);
- Metabolite sets associated with SNPs (currently contains 4500 entries);
- Predicted metabolite sets based on computational enzyme knockout model (currently contains 912 entries);
- Metabolite sets based on locations (currently contains 57 entries).

In addition, MSEA also allows user-defined metabolite sets to be uploaded to perform enrichment analysis on arbitrary groups of compounds which researchers want to test. The metabolite set library is simply a two-column comma separated text file with the first column for metabolite set names and the second column for its comound names (must use HMDB compound name) separated by ";". Please note, the built-in libraries are mainly from human studies. The functional grouping of metabolites may not be valid. Therefore, for data from subjects other than human being, users are suggested to upload their self-defined metabolite set libraries for enrichment analysis.

6. Enrichment Analysis

Over Representation Analysis (ORA) is performed when a list of compound names is provided. The list of compound list can be obtained through conventional feature selection methods, or from a clustering algorithm, or from the compounds with abnormal concentrations detected in SSP, to investigate if some biologically meaningful patterns can be identified.

ORA was implemented using the hypergeometric test to evaluate whether a particular metabolite set is represented more than expected by chance within the given compound list. One-tailed *p* values are provided after adjusting for multiple testing. Figure 1 and Table 2 below summarize the result.

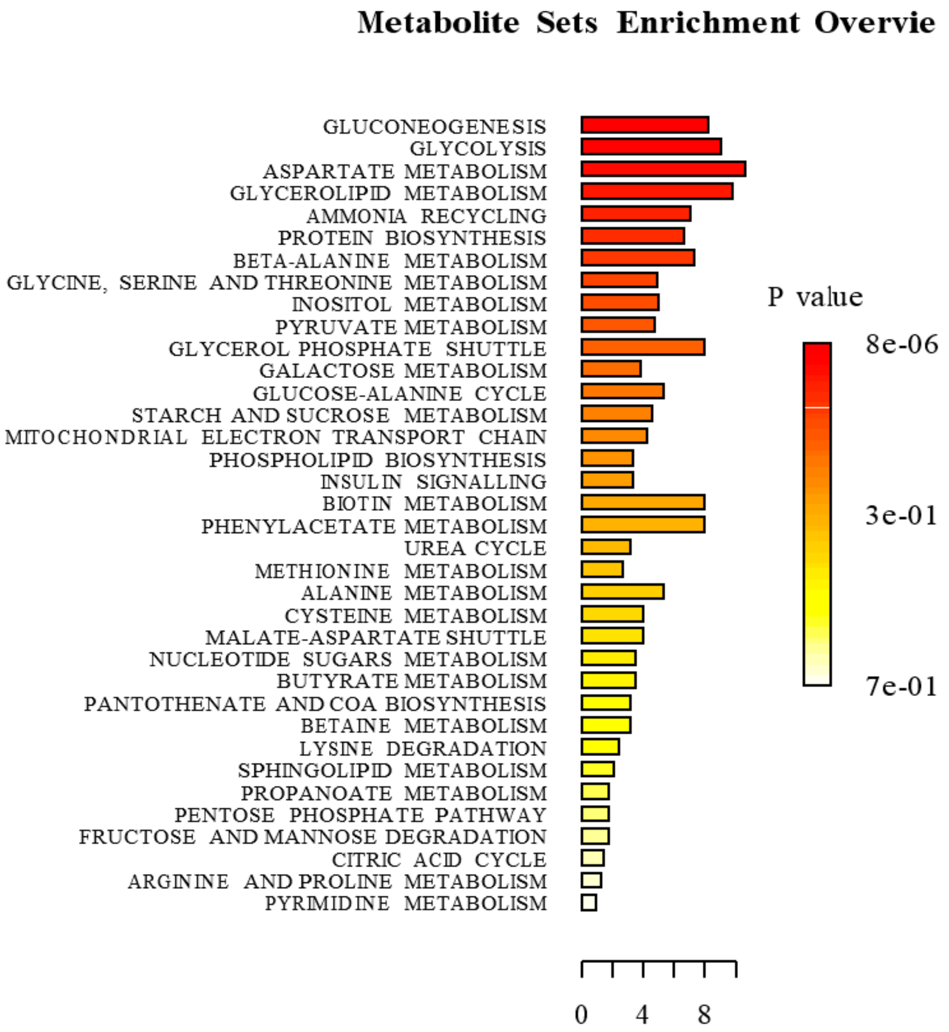

Figure 1. Summary Plot for Over Representation Analysis (ORA).

Table 2. Result from Over Representation Analysis.

Metabolite	Total	Expected	Hits	Raw *p*	Holm *p*	FDR
gluconeogenesis	27	0.85	7	7.68×10^{-6}	6.14×10^{-4}	6.14×10^{-4}
glycolysis	21	0.66	6	2.11×10^{-5}	1.67×10^{-3}	8.45×10^{-4}
aspartate metabolism	12	0.38	4	3.25×10^{-4}	2.53×10^{-2}	8.66×10^{-3}
glycerolipid metabolism	13	0.41	4	4.59×10^{-4}	3.53×10^{-2}	9.18×10^{-3}
ammonia recycling	18	0.57	4	1.76×10^{-3}	1.34×10^{-1}	2.82×10^{-2}
protein biosynthesis	19	0.60	4	2.18×10^{-3}	1.64×10^{-1}	2.91×10^{-2}
beta-alanine metabolism	13	0.41	3	6.45×10^{-3}	4.78×10^{-1}	7.24×10^{-2}
glycine, serine and threonine metabolism	26	0.82	4	7.24×10^{-3}	5.28×10^{-1}	7.24×10^{-2}
inositol metabolism	19	0.6	3	1.93×10^{-2}	1.00	1.71×10^{-1}
pyruvate metabolism	20	0.63	3	2.22×10^{-2}	1.00	1.73×10^{-1}
glycerol phosphate shuttle	8	0.25	2	2.38×10^{-2}	1.00	1.73×10^{-1}
galactose metabolism	25	0.79	3	4.03×10^{-2}	1.00	2.69×10^{-1}
glucose-alanine cycle	12	0.38	2	5.19×10^{-2}	1.00	3.20×10^{-1}
starch and sucrose metabolism	14	0.44	2	6.89×10^{-2}	1.00	3.94×10^{-1}
mitochondrial electron transport chain	15	0.47	2	7.80×10^{-2}	1.00	4.16×10^{-1}
phospholipid biosynthesis	19	0.6	2	1.18×10^{-1}	1.00	5.07×10^{-1}
insulin signalling	19	0.6	2	1.18×10^{-1}	1.00	5.07×10^{-1}
biotin metabolism	4	0.13	1	1.20×10^{-1}	1.00	5.07×10^{-1}

Table 2. *Cont.*

Metabolite	Total	Expected	Hits	Raw p	Holm p	FDR
phenylacetate metabolism	4	0.13	1	1.20×10^{-1}	1.00	5.07×10^{-1}
urea cycle	20	0.63	2	1.28×10^{-1}	1.00	5.13×10^{-1}
methionine metabolism	24	0.76	2	1.73×10^{-1}	1.00	6.37×10^{-1}
alanine metabolism	6	0.19	1	1.75×10^{-1}	1.00	6.37×10^{-1}
cysteine metabolism	8	0.25	1	2.27×10^{-1}	1.00	7.56×10^{-1}
malate-aspartate shuttle	8	0.25	1	2.27×10^{-1}	1.00	7.56×10^{-1}
nucleotide sugars metabolism	9	0.28	1	2.51×10^{-1}	1.00	7.74×10^{-1}
butyrate metabolism	9	0.28	1	2.51×10^{-1}	1.00	7.74×10^{-1}
pantothenate and coa biosyn-thesis	10	0.32	1	2.75×10^{-1}	1.00	7.87×10^{-1}
betaine metabolism lysine	10	0.32	1	2.75×10^{-1}	1.00	7.87×10^{-1}
degradation sphingolipid	13	0.41	1	3.43×10^{-1}	1.00	9.45×10^{-1}
metabolism propanoate	15	0.47	1	3.84×10^{-1}	1.00	1.00
metabolism pentose	18	0.57	1	4.42×10^{-1}	1.00	1.00
phosphate pathway fructose	18	0.57	1	4.42×10^{-1}	1.00	1.00
and mannose degra-dation citric acid cycle	18	0.57	1	4.42×10^{-1}	1.00	1.00
arginine and proline	23	0.72	1	5.26×10^{-1}	1.00	1.00
metabolism	26	0.82	1	5.71×10^{-1}	1.00	1.00
pyrimidine metabolism	36	1.13	1	6.92×10^{-1}	1.00	1.00

Section 3. MSEA Reports after Exposing MDA-MB-231 Cells to IC20 of Test Compounds

Metabolomic Data Analysis with MetaboAnalyst 3.0
User ID: guest845625042160926098
15 February 2016

1. Background

MESA or Metabolite Set Enrichment Analysis is a way to identify biologically meaningful patterns that are significantly enriched in quantitative metabolomic data. In conventional approaches, metabolites are evaluated individually for their significance under conditions of study. Those compounds that have passed certain sigificance level are then combined to see if any meaningful patterns can be discerned. In contrast, MSEA directly investigates if a set of functionally related metabolites without the need to preselect compounds based on some arbituary cut-off threshold. It has the potentail to identify subtle but consistent changes among a group of related compounds, which may go undetected with the conventional approaches.

Essentially, MSEA is a metabolomic version of the popular GSEA (Gene Set Enrichment Analysis) software with its own collection of metabolite set libraries as well as an implementation of user-friendly web-interfaces. GSEA is widely used in genomics data analysis and has proven to be a powerful alternative to conventional approaches. For more information, please refer to the original paper by Subramanian A, and a nice review paper by Nam D., Kim S.Y.

2. MSEA Overview

Metabolite set enrichment analysis consists of four steps—data input, data processing, data analysis, and results download. Different analysis procedures are performed based on different input types. In addition, users can also browse and search the metabolite set libraries as well as upload their self-defined metabolite sets for enrichment analysis. Users can also perform metabolite name mapping between a variety of compound names, synonyms, and major database identifiers.

3. Data Input

There are three enrichment analysis algorithms offered by MSEA. Accordingly, three different types of data inputs are required by these three approaches:

- A list of important compound names—entered as a one column data (Over Representation Analysis (ORA));

- A single measured biofluid (urine, blood, CSF) sample—entered as tab separated two-column data with the first column for compound name, and the second for concentration values (Single Sample Profiling (SSP));
- A compound concentration table-entered as a comma separated (.csv) file with the each sample per row and each metabolite concentration per column. The first column is sample names and the second column for sample phenotype labels (Quantitative Enrichment Analysis (QEA)).

You selected Over Representation Analysis (ORA) which requires a list of compound names as input.

4. Data Process

The first step is to standardize the compound labels. It is an essential step since the compound labels will be subsequently compared with compounds contained in the metabolite set library. MSEA has a built-in tool to convert between compound common names, synonyms, identifiers used in HMDB ID, PubChem, ChEBI, BiGG, METLIN, KEGG, or Reactome. Table 1 shows the conversion results. Note: 1 indicates exact match, 2 indicates approximate match, and 0 indicates no match. A text file contain the result can be found the downloaded file name map.csv.

Table 1. Result from Compound Name Mapping.

Number	Query	Match	HMDB	PubChem	KEGG	Comment
1	2-Oxoglutarate	Oxoglutaric acid	HMDB00208	51	C00026	1
2	6-Phosphogluconate	6-Phosphogluconic acid	HMDB01316	91493	C00345	1
3	L-Alanine	L-Alanine	HMDB00161	5950	C00041	1
4	β-Alanine	Beta-Alanine	HMDB00056	239	C00099	1
5	Cystathionine	L-Cystathionine	HMDB00099	439258	C02291	1
6	Dihydroxyacetone Phosphate	Dihydroxyacetone phosphate	HMDB01473	668	C00111	1
7	Fructose	D-Fructose	HMDB00660	439709	C02336	1
8	Glucose	D-Glucose	HMDB00122	5793	C00031	1
9	L-Glutamine	L-Glutamine	HMDB00641	5961	C00064	1
10	Glycerol 3-phosphate	Glycerol 3-phosphate	HMDB00126	439162	C00093	1
11	Hypotaurine	Hypotaurine	HMDB00965	107812	C00519	1
12	L-Leucine	L-Leucine	HMDB00687	6106	C00123	1
13	*N*-Acetylaspartate	*N*-Acetyl-L-aspartic acid	HMDB00812	65065	C01042	1
14	Phosphoenolpyruvate	Phosphoenolpyruvic acid	HMDB00263	1005	C00074	1
15	Putrescine	Putrescine	HMDB01414	1045	C00134	1
16	L-Serine	L-Serine	HMDB00187	5951	C00065	1
17	Succinate	Succinic acid	HMDB00254	1110	C00042	1
18	Tagatose	D-Tagatose	HMDB03418	92092	C00795	1
19	L-Valine	L-Valine	HMDB00883	6287	C00183	1

The second step is to check concentration values. For SSP analysis, the concentration must be measured in umol for blood and CSF samples. The urinary concentrations must be first converted to umol/mmol creatinine in order to compare with reported concentrations in literature. No missing or negative values are allowed in SSP analysis. The concentration data for QEA analysis is more flexible. Users can upload either the original concentration data or normalized data. Missing or negative val-ues are allowed (coded as NA) for QEA. Please note, MSEA does not perform data normalization. If normalization is important, you should first normalize your data before upload. You can use our compan-ion website MetaboAnalyst www.metaboanalyst.ca for a variety of data processing and normalization methods.

5. Selection of Metabolite Set Library

Before proceeding to enrichment analysis, a metabolite set library has to be chosen. There are seven built-in libraries offered by MSEA:

- Metabolic pathway associated metabolite sets (currently contains 88 entries);
- Disease associated metabolite sets (reported in blood) (currently contains 416 entries);

- Disease associated metabolite sets (reported in urine) (currently contains 346 entries);
- Disease associated metabolite sets (reported in CSF) (currently contains 124 entries);
- Metabolite sets associated with SNPs (currently contains 4500 entries);
- Predicted metabolite sets based on computational enzyme knockout model (currently contains 912 entries);
- Metabolite sets based on locations (currently contains 57 entries).

In addition, MSEA also allows user—defined metabolite sets to be uploaded to perform enrichment analysis on arbitrary groups of compounds which researchers want to test. The metabolite set library is simply a two-column comma separated text file with the first column for metabolite set names and the second column for its comound names (must use HMDB compound name) separated by ";". Please note, the built-in libraries are mainly from human studies. The functional grouping of metabolites may not be valid. Therefore, for data from subjects other than human being, users are suggested to upload their self-defined metabolite set libraries for enrichment analysis.

6. Enrichment Analysis

Over Representation Analysis (ORA) is performed when a list of compound names is provided. The list of compound list can be obtained through conventional feature selection methods, or from a clustering algorithm, or from the compounds with abnormal concentrations detected in SSP, to investigate if some biologically meaningful patterns can be identified.

ORA was implemented using the hypergeometric test to evaluate whether a particular metabolite set is represented more than expected by chance within the given compound list. One-tailed p values are provided after adjusting for multiple testing. Figure 1 and Table 2 below summarize the result.

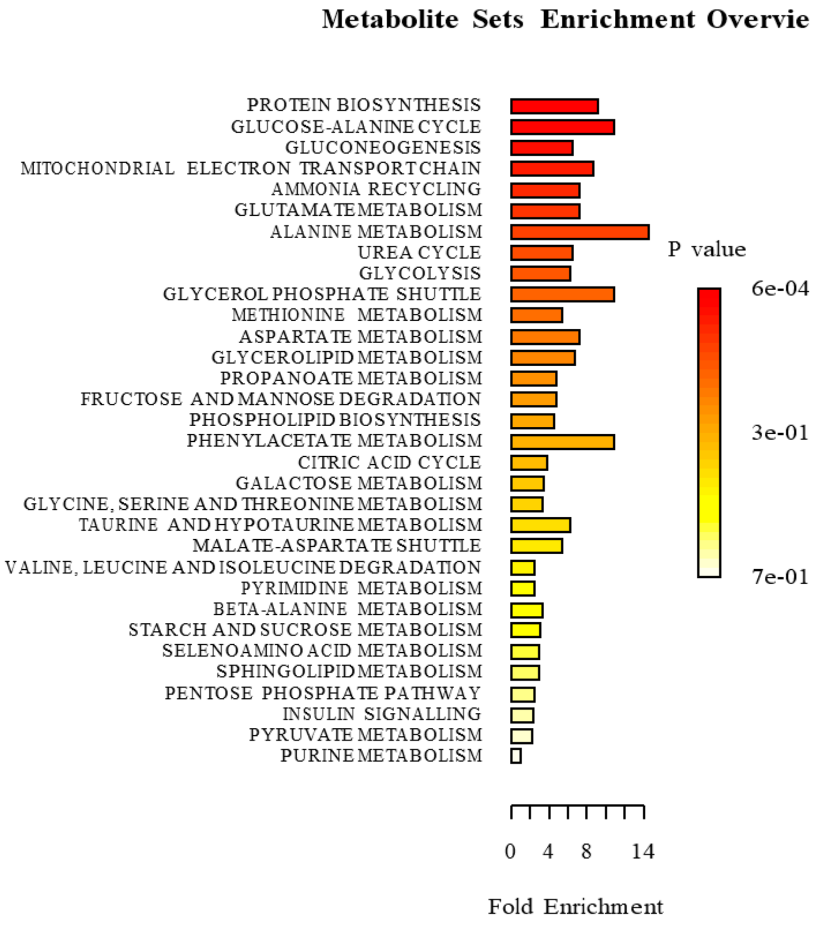

Figure 1. Summary Plot for Over Representation Analysis (ORA).

Table 2. Result from Over Representation Analysis.

Metabolite	Total	Expected	Hits	Raw *p*	Holm *p*	FDR
protein biosynthesis	19	0.44	4	6.28×10^{-4}	5.03×10^{-2}	5.03×10^{-2}
glucose-alanine cycle	12	0.28	3	2.00×10^{-3}	1.58×10^{-1}	6.74×10^{-2}
gluconeogenesis	27	0.62	4	2.53×10^{-3}	1.97×10^{-1}	6.74×10^{-2}
mitochondrial electron transport chain	15	0.34	3	3.96×10^{-3}	3.05×10^{-1}	7.93×10^{-2}
ammonia recycling	18	0.41	3	6.80×10^{-3}	5.17×10^{-1}	8.16×10^{-2}
glutamate metabolism	18	0.41	3	6.80×10^{-3}	5.17×10^{-1}	8.16×10^{-2}
alanine metabolism	6	0.14	2	7.14×10^{-3}	5.28×10^{-1}	8.16×10^{-2}
urea cycle	20	0.46	3	9.23×10^{-3}	6.74×10^{-1}	9.23×10^{-2}
glycolysis	21	0.48	3	1.06×10^{-2}	7.64×10^{-1}	9.43×10^{-2}
glycerol phosphate shuttle	8	0.18	2	1.30×10^{-2}	9.20×10^{-1}	1.04×10^{-1}
methionine metabolism	24	0.55	3	1.54×10^{-2}	1.00	1.12×10^{-1}
aspartate metabolism	12	0.28	2	2.89×10^{-2}	1.00	1.93×10^{-1}
glycerolipid metabolism	13	0.3	2	3.37×10^{-2}	1.00	2.07×10^{-1}
propanoate metabolism	18	0.41	2	6.17×10^{-2}	1.00	3.29×10^{-1}
fructose and mannose degra-dation	18	0.41	2	6.17×10^{-2}	1.00	3.29×10^{-1}
phospholipid biosynthesis	19	0.44	2	6.81×10^{-2}	1.00	3.40×10^{-1}
phenylacetate metabolism	4	0.09	1	8.91×10^{-2}	1.00	4.20×10^{-1}
citric acid cycle	23	0.53	2	9.54×10^{-2}	1.00	4.24×10^{-1}
galactose metabolism	25	0.58	2	1.10×10^{-1}	1.00	4.63×10^{-1}
glycine, serine and threonine metabolism	26	0.6	2	1.18×10^{-1}	1.00	4.71×10^{-1}
taurine and hypotaurine metabolism	7	0.16	1	1.51×10^{-1}	1.00	5.75×10^{-1}
malate-aspartate shuttle	8	0.18	1	1.71×10^{-1}	1.00	6.21×10^{-1}
valine, leucine and isoleucine degradation	36	0.83	2	1.99×10^{-1}	1.00	6.64×10^{-1}
pyrimidine metabolism beta	36	0.83	2	1.99×10^{-1}	1.00	6.64×10^{-1}
alanine metabolism starch	13	0.3	1	2.63×10^{-1}	1.00	8.42×10^{-1}
and sucrose metabolism	14	0.32	1	2.80×10^{-1}	1.00	8.49×10^{-1}
selenoamino acid metabolism	15	0.34	1	2.97×10^{-1}	1.00	8.49×10^{-1}
sphingolipid metabolism	15	0.34	1	2.97×10^{-1}	1.00	8.49×10^{-1}
pentose phosphate pathway	18	0.41	1	3.45×10^{-1}	1.00	9.53×10^{-1}
insulin signalling	19	0.44	1	3.61×10^{-1}	1.00	9.62×10^{-1}
pyruvate metabolism	20	0.46	1	3.76×10^{-1}	1.00	9.70×10^{-1}
purine metabolism	45	1.04	1	6.60×10^{-1}	1.00	1.00

Table S1. The metabolite consumption or release rate/cell after exposing MCF-7 and MDA-MB-231 cells to genistein, daidzein or the soy seed extract. The negative and positive values obtained after normalization indicate a net consumption or release, respectively, of the corresponding metabolite. The results represent the average of five biological replicates. The most significant features compared to control are marked in bold and were selected based on multiple *t*-test (α = 0.01) and a FC threshold of 1.5.

Name	MCF-7 Cells																		
	$\Delta = (C_{72\,h} - C_{t0})$/Cell Number$_{72\,h}$ (Equation (1))																		
	Control	Genistein						Daidzein						Extract					
		for IC20	*p*-Value	FC	for SC20	*p*-Value	FC	for IC20	*p*-Value	FC	for SC20	*p*-Value	FC	for IC20	*p*-Value	FC	for SC20	*p*-Value	FC
alanine	0.053	0.064	<0.0001	1.211	0.045	0.0018	0.856	0.055	0.2924	1.045	0.0398	<0.0001	0.7535	0.057	0.0065	1.090	0.039	<0.0001	0.742
arginine	0.006	0.002	0.3276	0.271	0.004	0.5897	0.727	−0.001	0.0626	0.186	0.0013	0.1277	0.2252	−0.002	0.0438	0.420	0.001	0.1132	0.173
asparagine	−0.010	−0.008	0.3077	0.827	−0.009	0.5976	0.911	−0.005	0.0642	0.477	−0.0089	0.6332	0.9000	−0.005	0.0714	0.545	−0.008	0.2201	0.803
aspartate	0.018	0.030	0.0447	1.621	0.023	0.1564	1.234	0.032	**0.0067**	**1.771**	0.0225	0.3349	1.2289	0.027	0.0915	1.497	0.021	0.5990	1.126
choline	−0.002	−0.002	0.0119	1.185	−0.002	0.2273	0.962	−0.002	0.0052	1.175	−0.0019	0.2667	0.9663	−0.002	0.1028	1.069	−0.002	0.0872	0.918
cystine	−0.015	−0.020	0.0021	1.368	−0.015	0.8473	0.984	−0.018	0.0290	1.195	−0.0137	0.4402	0.9296	−0.021	0.0003	1.446	−0.013	0.0580	0.848
formate	0.005	0.009	**0.0002**	**1.786**	0.005	0.5360	0.932	0.010	**<0.0001**	**1.871**	0.0054	0.7168	1.0328	0.014	**<0.0001**	**2.629**	0.005	0.5330	0.947
fructose	−0.005	−0.008	0.0179	1.853	−0.005	0.7205	1.107	−0.006	0.4216	1.301	−0.0041	0.7473	0.9022	−0.011	**0.0009**	**2.436**	−0.006	0.0768	1.358
fumarate	0.000	0.001	0.0334	1.634	0.000	0.1738	0.768	0.000	0.6482	1.104	0.0002	0.3435	0.7617	0.000	0.1622	0.777	0.000	0.5018	0.872
glucose	−0.594	−0.508	0.0066	0.855	−0.614	0.3578	1.033	−0.511	0.0155	0.859	−0.6079	0.5378	1.0233	−0.505	0.0103	0.850	−0.603	0.7191	1.015
glutamate	0.010	0.012	0.4062	1.185	0.011	0.4776	1.097	0.012	0.4719	1.210	0.0091	0.7545	0.9354	0.011	0.6582	1.110	0.008	0.1819	0.834
glutamine	−0.111	−0.112	0.8453	1.010	−0.113	0.8347	1.018	−0.107	0.5058	0.962	−0.1015	0.4364	0.9157	−0.111	0.9540	1.004	−0.100	0.0473	0.906
glycine	−0.002	−0.003	0.5539	1.521	−0.004	0.3657	1.767	−0.005	0.1163	2.451	−0.0067	0.0433	3.3036	0.005	0.0199	2.451	−0.007	**0.0086**	**3.386**
histidine	−0.005	−0.005	0.3134	1.119	−0.005	0.6247	0.953	−0.005	0.3822	1.108	−0.0046	0.5845	0.9468	−0.005	0.7746	1.035	−0.004	0.3705	0.930
hydroxy proline	−0.002	−0.003	0.3793	1.329	−0.002	0.8581	1.053	−0.001	0.2126	0.598	−0.0023	0.9668	1.0060	−0.001	0.1102	0.570	−0.002	0.3249	0.695
isoleucine	−0.030	−0.036	0.0080	1.181	−0.028	0.0561	0.917	−0.032	0.1507	1.062	−0.0278	0.0590	0.9133	−0.033	0.1157	1.088	−0.026	0.0029	0.850
lactate	0.606	0.941	**<0.0001**	**1.551**	0.622	0.5587	1.026	0.916	**<0.0001**	**1.510**	0.6836	0.0126	1.1274	0.827	<0.0001	1.363	0.667	0.0147	1.101
leucine	−0.043	−0.052	0.0016	1.217	−0.039	0.0140	0.912	−0.048	0.0073	1.113	−0.0394	0.0227	0.9218	−0.047	0.0960	1.102	−0.037	0.0006	0.857
lysine	−0.035	−0.036	0.7662	1.013	−0.033	0.0289	0.925	−0.034	0.4684	0.974	−0.0334	0.1214	0.9472	−0.032	0.1954	0.921	−0.031	0.0093	0.879
methionine	−0.006	−0.002	0.0903	0.337	−0.006	0.6781	0.932	−0.005	0.3765	0.818	−0.0059	0.9997	0.9981	−0.005	0.5479	0.828	−0.005	0.5214	0.880
myo-inositol	−0.003	−0.005	**0.0047**	**1.786**	−0.003	0.6329	1.102	−0.004	0.0236	1.508	−0.0038	0.2190	1.3847	−0.005	**0.0086**	**1.982**	−0.003	0.3411	1.182
ornithine	0.007	0.013	**<0.0001**	**1.902**	0.007	0.5350	1.051	0.012	**0.0004**	**1.699**	0.0073	0.6718	1.0536	0.013	**<0.0001**	**1.838**	0.007	0.7767	0.978
oxoglutarat	0.002	0.003	0.1262	1.503	0.003	0.1422	1.251	0.002	0.2524	1.239	0.0020	0.9275	0.9840	0.001	0.0462	0.569	0.002	0.2297	0.806
phenylalani	−0.009	−0.009	0.5538	1.041	−0.008	0.3952	0.954	−0.009	0.4819	1.049	−0.0077	0.0957	0.9014	−0.009	0.8411	1.018	−0.008	0.1448	0.929
ne proline	0.005	0.014	**0.0014**	**2.720**	0.007	0.3305	1.315	0.015	**<0.0001**	**2.897**	0.0085	0.0651	1.6400	0.013	**<0.0001**	**2.578**	0.009	0.0198	1.769
pyruvate	−0.009	−0.030	**<0.0001**	**3.451**	−0.011	0.0146	1.331	−0.032	**<0.0001**	**3.692**	−0.0212	**<0.0001**	**2.4709**	−0.034	**<0.0001**	**4.005**	−0.019	**<0.0001**	**2.217**
serine	−0.008	−0.014	0.0227	1.725	−0.010	0.2623	1.198	−0.016	**0.0031**	**2.027**	−0.0115	0.0509	1.4267	−0.017	**0.0009**	**2.084**	−0.011	0.1123	1.329
succinate	0.001	0.000	0.0412	0.252	0.000	0.0236	0.391	0.000	0.0559	0.378	0.0002	**0.0078**	**0.2050**	0.000	**0.0081**	**0.341**	0.000	**0.0038**	**0.395**
threonine	−0.011	−0.011	0.2470	0.949	−0.010	0.0051	0.858	−0.010	0.2949	0.942	−0.0099	0.0244	0.8887	−0.009	0.0240	0.828	−0.009	0.0004	0.793
tyrosine	−0.007	−0.008	0.0986	1.140	−0.007	0.4806	0.961	−0.007	0.8445	0.990	−0.0067	0.4346	0.9625	−0.007	0.5361	0.948	−0.006	0.1418	0.925
valine	−0.016	−0.017	0.0347	1.076	−0.015	0.0608	0.925	−0.018	0.0092	1.118	−0.0150	0.1131	0.9447	−0.017	0.3961	1.049	−0.014	0.0025	0.874

Table S1. *Cont.*

Name	MDA-MB-231 Cells									
	Δ = ($C_{72\,h}$ − C_{t0})/Cell Number$_{72\,h}$ (Equation (1))									
	Control	Genistein			Daidzein			Extract		
		IC20	*p*-Value	FC	IC20	*p*-Value	FC	IC20	*p*-Value	FC
alanine	0.0359	0.043	0.0650	1.185	0.045	0.0205	1.266	0.041	0.0837	1.154
arginine	−0.0070	−0.007	0.9856	0.999	−0.007	0.9305	1.012	−0.006	0.3679	0.810
asparagine	−0.0132	−0.013	0.8766	1.013	−0.016	0.0403	1.193	−0.014	0.7490	1.046
aspartate	0.0288	0.034	0.1480	1.191	0.037	0.1337	1.271	0.034	0.2480	1.163
choline	−0.0003	0.000	0.8383	1.160	0.000	0.3670	0.473	0.000	0.3708	0.555
cystine	−0.0081	−0.010	0.4822	1.250	−0.011	0.3178	1.331	−0.010	0.5158	1.239
formate	0.0056	0.006	0.0979	1.145	0.009	**<0.0001**	**1.570**	0.008	0.0002	1.390
fructose	−0.0093	−0.012	0.1184	1.278	−0.012	0.0727	1.308	−0.014	**0.0042**	**1.547**
fumarate	0.0000	0.000	0.3706	0.501	0.000	0.3972	2.348	0.000	0.1151	2.036
glucose	−0.8973	−0.819	0.0072	0.912	−0.840	0.0474	0.936	−0.817	0.0047	0.910
glutamate	0.0155	0.016	0.4650	1.065	0.018	0.1193	1.169	0.017	0.4386	1.101
glutamine	−0.1062	−0.092	0.0723	0.870	−0.093	0.0932	0.875	−0.088	0.0375	0.829
glycine	−0.0063	−0.006	0.8246	0.941	−0.005	0.5553	0.776	−0.005	0.5618	0.814
histidine	−0.0048	−0.005	0.8936	0.992	−0.005	0.9667	1.004	−0.005	0.6503	1.045
hydroxyproline	−0.0019	−0.003	0.1331	1.692	−0.003	0.2151	1.602	−0.003	0.0825	1.629
isoleucine	−0.0247	−0.029	0.0002	1.187	−0.031	<0.0001	1.235	−0.029	<0.0001	1.192
lactate	1.1739	1.262	0.0264	1.075	1.385	0.0001	1.180	1.289	0.0179	1.098
leucine	−0.0352	−0.040	0.0028	1.132	−0.041	0.0014	1.167	−0.041	0.0016	1.163
lysine	−0.0267	−0.026	0.8285	0.987	−0.026	0.7383	0.975	−0.026	0.5440	0.959
methionine	−0.0062	−0.003	0.0979	0.495	−0.004	0.1454	0.608	−0.003	0.0327	0.529
myo-inositol	−0.0059	−0.007	0.4864	1.144	−0.007	0.2980	1.236	−0.007	0.3634	1.206
ornithine	0.0277	0.034	<0.0001	1.224	0.034	<0.0001	1.238	0.034	<0.0001	1.237
oxoglutarat	0.0016	0.002	0.3877	1.155	0.001	0.2321	0.744	0.001	**0.0083**	**0.419**
phenylalanine	−0.0056	−0.006	0.8331	1.017	−0.006	0.2096	1.076	−0.006	0.9580	1.006
proline	0.0001	−0.001	0.2163	7.436	−0.001	0.5406	3.914	0.000	0.4491	3.393
pyruvate	−0.0463	−0.053	0.0634	1.140	−0.055	0.0111	1.197	−0.054	0.0235	1.173
serine	−0.0106	−0.009	0.6165	0.864	−0.012	0.5843	1.143	−0.011	0.7499	1.075
succinate	−0.0006	−0.001	0.1470	1.496	−0.001	**0.0076**	**2.194**	−0.001	0.2011	1.465
threonine	−0.0092	−0.009	0.7030	1.036	−0.010	0.4807	1.062	−0.009	0.9635	1.009
tyrosine	−0.0051	−0.006	0.4703	1.106	−0.006	0.3604	1.124	−0.006	0.5696	1.085
valine	−0.0136	−0.015	0.0213	1.076	−0.015	0.0259	1.083	−0.015	0.0226	1.086

Δ = the metabolite consumption or release rate/cell; $C_{72\,h}$ = the relative concentration of metabolite after 72 h of treatment; C_{t0} = the relative concentration of metabolite in initial medium (t_0); cell number$_{72\,h}$ = the cell number after 72 h of treatment.

Table S2. The concentration of intracellular metabolites identified after exposing MCF-7 and MDA-MB-231 brest cancer cells to genistein, daidzein and the soy seed extract for 72h. The results represent the average of five biological replicates. For each metabolite, the relative concentration was normalized to the yielded cell number in order to obtain the relative amount per cell. The most significant features compared to control are marked in bold and were selected based on multiple *t*-test (α = 0.01) and a FC threshold of 1.5.

Name	MCF-7 Cells																		
	$C_{normalized} = C_{relative}/\text{Cell Number}_{72\ h}$																		
	Control	Genistein						Daidzein						Extract					
		IC20	*p*-Value	FC	SC20	*p*-Value	FC	IC20	*p*-Value	FC	SC20	*p*-Value	FC	IC20	*p*-Value	FC	SC20	*p*-Value	FC
1-methyl nicotinamide	0.017	0.023	0.0996	1.349	0.015	0.4856	0.892	0.024	0.0569	1.437	0.021	0.1967	1.237	0.027	0.0601	1.598	0.017	0.7955	1.028
2-hydroxypyridine	0.014	0.019	0.0466	1.341	0.015	0.7912	1.036	0.018	0.2020	1.273	0.016	0.5383	1.110	0.023	0.0316	1.586	0.016	0.5054	1.126
2-oxoglutarate	0.026	0.020	0.0686	0.780	0.023	0.3313	0.897	0.022	0.1091	0.856	0.025	0.9356	0.982	0.017	0.0121	0.675	0.023	0.4375	0.877
3-hydroxybutyrate	0.001	0.001	**0.0002**	**1.622**	0.001	0.4258	1.059	0.001	0.0016	1.474	0.001	0.0244	1.185	0.001	0.0011	1.379	0.001	0.0703	1.203
3-methyl-2-oxovalerate	0.005	0.006	0.0074	1.322	0.004	0.4427	0.924	0.005	0.1416	1.161	0.004	0.1749	0.869	0.004	0.5652	0.940	0.004	0.1369	0.843
3-phosphoglycerate	0.039	0.026	**0.0011**	**0.657**	0.022	**0.0005**	**0.575**	0.025	**0.0010**	**0.645**	0.020	**0.0002**	**0.502**	0.015	**<0.0001**	**0.394**	0.022	**0.0003**	**0.553**
4-guanidinobutyrate	0.009	0.010	0.2520	1.108	0.006	**0.0002**	**0.639**	0.014	0.0010	1.446	0.007	0.0435	0.789	0.013	0.0036	1.332	0.008	0.1119	0.796
4-hydroxyproline	1.503	1.118	0.0420	0.744	1.312	0.2435	0.873	1.096	0.0408	0.729	1.364	0.3984	0.908	0.877	**0.0026**	**0.583**	1.459	0.8583	0.971
4-methyl-2-oxovalerate	0.005	0.007	0.0014	1.475	0.004	0.9235	0.984	0.006	0.0748	1.224	0.004	0.4641	0.905	0.005	0.6745	1.043	0.004	0.2101	0.847
5-oxoproline	0.615	0.830	0.0013	1.350	0.567	0.2473	0.922	0.802	0.0036	1.306	0.627	0.7071	1.020	0.847	0.0002	1.378	0.608	0.9470	0.990
6-phosphogluconate	0.074	0.058	0.0512	0.781	0.092	0.0428	1.244	0.058	0.0472	0.786	0.074	0.8156	1.005	0.063	0.2036	0.860	0.106	0.0042	1.439
acetamide	0.081	0.097	0.6928	1.204	0.089	0.7157	1.099	0.115	0.2232	1.421	0.123	0.2686	1.524	0.126	0.1731	1.553	0.097	0.5694	1.195
adenine	0.023	0.029	0.0553	1.246	0.019	0.2546	0.821	0.029	0.0226	1.267	0.022	0.5641	0.936	0.030	0.0633	1.291	0.022	0.6793	0.957
alanine	2.206	1.473	0.0150	0.668	1.861	0.2072	0.844	1.281	**0.0040**	**0.581**	1.732	0.0683	0.785	0.912	**0.0002**	**0.413**	1.919	0.3221	0.870
aminomalonate	0.179	0.125	0.3536	0.696	0.180	0.9826	1.002	0.138	0.4491	0.768	0.143	0.4697	0.796	0.169	0.9019	0.943	0.159	0.7754	0.889
arabinofuranose	0.027	0.029	0.4411	1.079	0.026	0.8044	0.958	0.026	0.8344	0.972	0.028	0.7986	1.026	0.024	0.4540	0.876	0.023	0.3409	0.860
asparagine	0.604	0.469	0.1325	0.777	0.534	0.3913	0.885	0.450	0.0982	0.746	0.593	0.8298	0.982	0.354	**0.0053**	**0.587**	0.632	0.7643	1.047
aspartate	0.579	0.322	**0.0026**	**0.555**	0.440	0.0503	0.759	0.254	**0.0003**	**0.439**	0.278	**0.0006**	**0.480**	0.330	**0.0021**	**0.570**	0.322	**0.0028**	**0.557**
β-alanine	0.004	0.007	**0.0064**	**1.712**	0.005	0.5470	1.079	0.007	0.0108	1.497	0.006	0.0124	1.477	0.005	0.1356	1.172	0.007	**0.0042**	**1.618**
butane	0.015	0.019	0.0142	1.275	0.015	0.7841	1.018	0.018	0.0205	1.214	0.015	0.7699	1.020	0.016	0.2186	1.101	0.015	0.8765	1.003
butyrate	0.002	0.002	0.0187	1.455	0.002	0.4723	1.094	0.002	**0.0050**	**1.511**	0.002	0.2290	1.157	0.003	0.0297	1.829	0.002	0.7154	1.039
cholesterol	0.001	0.002	0.4626	2.245	0.003	0.1927	2.532	0.001	0.7949	1.397	0.001	0.5786	0.665	0.001	0.8541	0.910	0.002	0.3853	2.352
citrate	0.437	0.564	0.0058	1.291	0.436	0.9819	0.999	0.524	0.0336	1.198	0.421	0.6297	0.964	0.490	0.1624	1.122	0.430	0.8667	0.984
citrulline	0.016	0.017	0.6678	1.094	0.016	0.9718	0.994	0.017	0.8446	1.045	0.019	0.5355	1.175	0.016	0.9710	1.004	0.021	0.2881	1.354
creatine	0.009	0.011	0.3023	1.215	0.008	0.6059	0.921	0.010	0.4981	1.169	0.009	0.9279	1.002	0.010	0.6649	1.090	0.008	0.7439	0.938
cystathionine	0.057	0.035	0.1085	0.611	0.044	0.3884	0.772	0.024	0.0342	0.423	0.026	0.0521	0.462	0.018	0.0130	0.318	0.028	0.0581	0.487
cysteine	0.025	0.021	0.6513	0.854	0.026	0.8731	1.047	0.030	0.6020	1.185	0.035	0.2020	1.414	0.025	0.9885	1.004	0.039	0.1518	1.568
cysteinylglycine	0.007	0.008	0.9522	1.073	0.004	0.4589	0.545	0.009	0.7641	1.323	0.009	0.7648	1.236	0.010	0.7350	1.401	0.007	0.9731	1.060
D-glucose-6-phosphate	0.014	0.011	0.2026	0.780	0.018	0.1642	1.312	0.011	0.3143	0.816	0.016	0.6510	1.126	0.006	**0.0096**	**0.421**	0.019	0.2097	1.335
dihydroxyacetone phosphate	0.007	0.012	**0.0021**	**1.614**	0.004	**0.0010**	**0.513**	0.011	**0.0015**	**1.476**	0.008	0.1235	1.156	0.008	0.1316	1.159	0.006	0.2935	0.876
D-mannitol	0.006	0.028	0.4187	4.281	0.021	0.3972	3.255	0.014	0.4812	2.104	0.004	0.6446	0.675	0.004	0.4846	0.579	0.003	0.3842	0.480
D-ribose-5-phosphate	0.005	0.005	0.5715	0.923	0.008	0.0135	1.497	0.005	0.7109	1.053	0.006	0.1432	1.209	0.005	0.7840	0.943	0.010	**0.0009**	**1.868**

Table S2. *Cont.*

Name	MCF-7 Cells																		
	**$C_{normalized} = C_{relative}$/Cell Number$_{72\ h}$**																		
	**Control**	**Genistein**						**Daidzein**						**Extract**					
		**IC20**	***p*-Value**	**FC**	**SC20**	***p*-Value**	**FC**	**IC20**	***p*-Value**	**FC**	**SC20**	***p*-Value**	**FC**	**IC20**	***p*-Value**	**FC**	**SC20**	***p*-Value**	**FC**
fructose	0.015	0.024	0.0118	1.549	0.018	0.1831	1.185	0.019	0.4113	1.239	0.019	0.2308	1.211	0.011	0.0561	0.734	0.017	0.3521	1.123
fructose-6-phosphate	0.007	0.005	0.2467	0.795	0.007	0.7515	1.063	0.006	0.7976	0.963	0.007	0.8318	1.067	0.004	0.0269	0.561	0.008	0.2746	1.243
fumarate	0.053	0.056	0.5456	1.065	0.039	0.1014	0.743	0.050	0.7901	0.949	0.038	0.0847	0.726	0.059	0.4794	1.118	0.032	0.0610	0.615
gluconate	0.015	0.032	**0.0097**	**2.109**	0.021	0.0721	1.391	0.007	0.0103	0.446	0.015	0.8365	0.975	0.002	**0.0007**	**0.137**	0.021	0.0597	1.383
glucose	0.174	0.113	**<0.0001**	**0.647**	0.211	0.0406	1.207	0.118	0.0003	0.678	0.185	0.3432	1.060	0.105	**<0.0001**	**0.600**	0.200	0.1533	1.147
glucuronate	0.006	0.008	0.0027	1.489	0.005	0.5445	0.940	0.010	**0.0002**	**1.731**	0.007	0.1275	1.203	0.010	**0.0066**	**1.767**	0.007	0.1075	1.201
glutamate	3.224	2.285	0.0009	0.709	2.852	0.0953	0.885	2.532	0.0059	0.785	2.947	0.2514	0.914	2.365	0.0024	0.734	3.092	0.5103	0.959
glutamine	0.065	0.055	0.2662	0.853	0.069	0.8265	1.059	0.059	0.3293	0.909	0.062	0.8671	0.953	0.054	0.2168	0.830	0.061	0.8450	0.931
glycerate	0.002	0.002	0.0078	0.732	0.002	0.0063	0.768	0.001	**<0.0001**	**0.630**	0.001	**<0.0001**	**0.562**	0.001	**<0.0001**	**0.461**	0.001	**<0.0001**	**0.555**
glycerol	0.019	0.063	0.0218	3.361	0.031	0.1641	1.663	0.055	0.0105	2.957	0.041	0.0185	2.211	0.047	0.0122	2.508	0.038	0.0418	2.040
glycerol 3-phosphate	0.217	0.432	**0.0004**	**1.993**	0.269	0.0827	1.243	0.490	**0.0002**	**2.264**	0.369	**0.0007**	**1.703**	0.499	**0.0002**	**2.302**	0.368	**0.0068**	**1.698**
glycine	2.281	1.726	0.0146	0.757	2.282	0.9935	1.001	1.858	0.0521	0.815	2.031	0.1440	0.890	2.167	0.5185	0.950	2.090	0.4557	0.916
glycolate	0.001	0.001	0.5303	1.534	0.000	0.3218	0.656	0.001	0.6265	1.268	0.001	0.9107	1.020	0.001	0.5426	1.430	0.001	0.9293	0.989
glycylglycine	0.024	0.017	0.2652	0.684	0.018	0.2954	0.742	0.032	0.3660	1.330	0.017	0.3281	0.696	0.031	0.4274	1.256	0.017	0.3994	0.720
hexanoate	0.001	0.001	0.0237	1.351	0.001	0.9293	1.024	0.001	0.6874	1.063	0.001	0.3331	0.857	0.001	0.2904	1.156	0.001	0.0832	0.739
histidine	0.024	0.020	0.3744	0.837	0.022	0.6354	0.939	0.015	0.0957	0.625	0.020	0.4447	0.848	0.016	0.1352	0.655	0.024	0.8719	1.022
hypotaurine	0.040	0.059	0.1169	1.481	0.045	0.5653	1.122	0.043	0.6440	1.084	0.040	0.9465	0.987	0.038	0.7784	0.951	0.044	0.6004	1.091
indole-2,3-dione	0.001	0.001	0.2057	0.780	0.001	0.7235	0.946	0.001	0.3250	0.842	0.001	0.2637	0.832	0.001	0.0790	0.688	0.001	0.9873	1.005
isocitrate	0.006	0.006	0.1789	1.116	0.006	0.8794	1.010	0.006	0.5830	1.042	0.005	0.5123	0.952	0.005	0.7354	0.975	0.006	0.8065	0.979
isoleucine	0.316	0.331	0.6216	1.045	0.281	0.3502	0.889	0.302	0.5723	0.955	0.336	0.5418	1.063	0.281	0.2040	0.887	0.342	0.4417	1.082
lactate	2.415	3.792	**<0.0001**	**1.570**	2.402	0.9934	0.994	3.569	0.0005	1.478	2.823	0.0321	1.169	2.754	0.0419	1.140	2.669	0.2807	1.105
leucine	0.268	0.321	0.1536	1.198	0.226	0.1884	0.842	0.273	0.8268	1.016	0.270	0.9515	1.007	0.271	0.8489	1.009	0.266	0.9893	0.992
lysine	0.019	0.062	**0.0014**	**3.277**	0.015	0.2598	0.785	0.035	0.0451	1.869	0.017	0.7973	0.895	0.058	**0.0004**	**3.063**	0.016	0.4474	0.864
malate	0.131	0.131	0.9789	1.001	0.099	0.0844	0.756	0.126	0.7883	0.965	0.090	0.0404	0.690	0.127	0.7558	0.973	0.094	0.0571	0.720
methionine	0.025	0.055	**0.0061**	**2.177**	0.016	0.2364	0.642	0.040	0.0985	1.561	0.016	0.3758	0.638	0.052	**0.0055**	**2.044**	0.013	0.1145	0.530
myo-inositole	1.389	3.486	**<0.0001**	**2.510**	1.503	0.5502	1.082	3.967	**<0.0001**	**2.856**	2.649	**0.0001**	**1.907**	5.655	**<0.0001**	**4.072**	2.479	**0.0009**	**1.785**
N-acetylaspartate	0.053	0.044	0.1070	0.842	0.042	0.0255	0.802	0.032	**0.0005**	**0.609**	0.032	**0.0001**	**0.608**	0.052	0.8325	0.979	0.037	0.0044	0.703
O-phosphocolamine	0.971	1.158	0.4525	1.193	1.014	0.8230	1.044	0.559	0.0341	0.576	0.388	**0.0065**	**0.399**	0.837	0.4561	0.862	0.448	0.0135	0.461
ornithine	0.100	0.134	0.0013	1.346	0.095	0.4970	0.950	0.117	0.0260	1.173	0.114	0.0160	1.147	0.094	0.2873	0.944	0.125	0.0062	1.256
pantothenate	0.012	0.014	0.2834	1.099	0.010	0.0442	0.829	0.014	0.0901	1.134	0.010	0.0381	0.828	0.022	**0.0003**	**1.797**	0.012	0.5828	0.947
phosphoenolpyruvate	0.008	0.005	**0.0001**	**0.653**	0.004	**<0.0001**	**0.591**	0.005	**<0.0001**	**0.657**	0.004	**<0.0001**	**0.531**	0.004	**<0.0001**	**0.513**	0.004	**<0.0001**	**0.563**
proline	2.418	2.608	0.5286	1.079	2.306	0.6915	0.954	3.134	0.0063	1.296	3.161	0.0035	1.307	3.298	0.0083	1.364	3.039	0.0283	1.257
putrescine	0.150	0.107	0.0068	0.717	0.091	**0.0003**	**0.610**	0.195	0.0868	1.305	0.107	0.0704	0.715	0.176	0.0701	1.174	0.090	**0.0023**	**0.600**
pyrophosphate	0.945	0.978	0.8788	1.035	0.960	0.8833	1.016	1.079	0.4186	1.141	1.043	0.4834	1.104	1.108	0.3521	1.172	0.986	0.8608	1.043
pyruvate	0.054	0.039	0.0149	0.724	0.043	0.0260	0.796	0.028	**0.0003**	**0.507**	0.029	**0.0003**	**0.534**	0.025	**0.0002**	**0.453**	0.028	**0.0003**	**0.517**
ribulose-5-phosphate	0.006	0.006	0.8230	0.975	0.008	0.0246	1.285	0.006	0.8103	0.966	0.008	0.0789	1.208	0.005	0.1142	0.826	0.010	0.0102	1.486
serine	0.480	0.297	**0.0048**	**0.619**	0.303	**0.0044**	**0.630**	0.201	**<0.0001**	**0.418**	0.200	**<0.0001**	**0.416**	0.183	**<0.0001**	**0.381**	0.197	**<0.0001**	**0.410**

Table S2. *Cont.*

Name	MCF-7 Cells																	
	Cnormalized = Crelative/Cell Number$_{72\ h}$																	
	Control	Genistein						Daidzein						Extract				
		IC20	*p*-Value	FC	SC20	*p*-Value	FC	IC20	*p*-Value	FC	SC20	*p*-Value	FC	IC20	*p*-Value	FC	SC20	*p*-Value	FC
spermidine	0.011	0.012	0.9196	1.088	0.009	0.8056	0.882	0.012	0.8645	1.138	0.009	0.9214	0.886	0.011	0.9714	1.003	0.007	0.4596	0.668
succinate	0.019	0.030	0.0131	1.598	0.018	0.8707	0.975	0.023	0.1944	1.241	0.014	0.2147	0.775	0.025	0.0444	1.341	0.015	0.2742	0.787
tagatose	0.013	0.021	**0.0064**	**1.598**	0.015	0.2359	1.161	0.016	0.3254	1.208	0.016	0.2228	1.204	0.010	0.0591	0.729	0.014	0.3768	1.091
threonate	0.069	0.091	0.0021	1.329	0.063	0.3415	0.912	0.106	**0.0002**	**1.552**	0.075	0.2297	1.088	0.105	**0.0004**	**1.528**	0.074	0.2972	1.084
threonine	0.079	0.094	0.1591	1.187	0.061	0.0507	0.766	0.074	0.5653	0.929	0.062	0.0725	0.784	0.079	0.9831	0.992	0.065	0.1300	0.824
tryptophan	0.023	0.036	0.0374	1.568	0.018	0.2424	0.784	0.024	0.7676	1.065	0.014	0.1465	0.604	0.034	0.0885	1.476	0.012	0.0315	0.537
tyrosine	0.361	0.387	0.3548	1.073	0.340	0.5636	0.942	0.335	0.4660	0.930	0.354	0.7938	0.982	0.320	0.1804	0.888	0.387	0.5337	1.075
urea	0.102	0.124	0.0208	1.213	0.099	0.7186	0.969	0.117	0.1195	1.145	0.101	0.8539	0.986	0.115	0.1011	1.125	0.101	0.9409	0.987
uridine 5′-monophosphate	0.021	0.026	0.4465	1.272	0.031	0.0902	1.508	0.024	0.6070	1.151	0.029	0.1788	1.423	0.031	0.1568	1.524	0.032	0.2164	1.530
valine	0.027	0.072	0.0212	2.709	0.013	0.1925	0.484	0.040	0.3242	1.502	0.013	0.4203	0.488	0.054	0.0487	2.043	0.003	0.0253	0.105
unknown 1	0.041	0.062	0.2754	1.499	0.037	0.7372	0.887	0.052	0.4597	1.269	0.040	0.9331	0.965	0.056	0.3661	1.347	0.036	0.7572	0.875
unknown 2	0.002	0.003	**0.0049**	**1.666**	0.003	0.0718	1.377	0.004	**0.0029**	**1.900**	0.003	0.0646	1.391	0.005	**0.0037**	**2.338**	0.004	**0.0037**	**1.770**

Table S2. *Cont.*

Name	MDA-MB-231 Cells									
	Cnormalized = Crelative/Cell Number$_{72\ h}$									
	Control	Genistein			Daidzein			Extract		
		IC20	*p*-Value	FC	IC20	*p*-Value	FC	IC20	*p*-Value	FC
1-methyl nicotinamide	0.010	0.007	0.0020	0.724	0.007	0.0206	0.758	0.007	0.0019	0.719
2-hydroxypyridine	0.038	0.030	0.0235	0.791	0.032	0.0757	0.861	0.036	0.5907	0.948
2-oxoglutarate	0.058	0.037	**0.0001**	**0.642**	0.041	0.0005	0.701	0.043	0.0019	0.740
3-hydroxybutyrate	0.001	0.001	0.0919	0.835	0.001	0.0317	0.769	0.001	0.1046	0.829
3-methyl-2-oxovalerate	0.003	0.003	0.2116	0.878	0.003	0.9131	0.995	0.003	0.1792	0.888
3-phosphoglycerate	0.011	0.009	0.0130	0.805	0.009	0.0796	0.856	0.009	0.0560	0.842
4-guanidinobutyrate	0.082	0.089	0.1894	1.079	0.084	0.6999	1.023	0.091	0.0931	1.100
4-hydroxyproline	0.168	0.133	0.0035	0.791	0.169	0.9462	1.007	0.166	0.7030	0.988
4-methyl-2-oxovalerate	0.004	0.004	0.0340	0.868	0.004	0.8000	0.980	0.004	0.3132	0.920
5-oxoproline	0.753	0.434	0.0206	0.576	0.588	0.0059	0.781	0.600	0.0102	0.796
6-phosphogluconate	0.004	0.002	**0.0010**	**0.652**	0.003	0.0021	0.707	0.003	0.0094	0.673
acetamide	0.024	0.040	0.0733	1.619	0.032	0.3726	1.321	0.025	0.9853	1.013
adenine alanine	0.013	0.011	0.0089	0.787	0.011	0.0108	0.791	0.012	0.1625	0.873
aminomalonate	0.131	0.160	0.0174	1.226	0.204	**0.0082**	**1.563**	0.179	0.0036	1.374
arabinofuranose	0.040	0.057	0.0619	1.440	0.059	0.0458	1.495	0.063	0.0267	1.586

Table S2. *Cont.*

Name	MDA-MB-231 Cells									
	Cnormalized = Crelative/Cell Number$_{72\ h}$									
	Control	Genistein			Daidzein			Extract		
		IC20	*p*-Value	FC	IC20	*p*-Value	FC	IC20	*p*-Value	FC
asparagine	0.026	0.023	0.0846	0.858	0.026	0.7428	0.974	0.026	0.8462	0.995
aspartate	0.054	0.043	0.0110	0.797	0.057	0.6344	1.051	0.053	0.7469	0.981
β-alanine	0.026	0.034	0.0692	1.317	0.034	0.0752	1.324	0.037	0.0289	1.420
butane	0.089	0.043	**0.0002**	**0.485**	0.036	**<0.0001**	**0.406**	0.048	**0.0009**	**0.540**
butyrate	0.021	0.015	0.0003	0.708	0.015	0.0004	0.715	0.016	0.0037	0.770
cholesterol	0.000	0.000	-	-	0.000	-	-	0.000	-	-
citrate	0.001	0.001	0.9224	1.083	0.002	0.2286	1.708	0.001	0.6368	1.250
citrulline	0.379	0.257	<0.0001	0.678	0.266	<0.0001	0.701	0.285	0.0019	0.751
creatine	0.001	0.001	0.6414	0.934	0.002	0.0409	1.293	0.001	0.2955	1.150
cystathionine	0.012	0.009	0.0047	0.726	0.008	0.0112	0.714	0.009	0.0185	0.725
cysteine	0.002	0.002	0.5735	1.121	0.004	**0.0054**	**1.867**	0.003	0.0441	1.525
cysteinylglycine	0.002	0.002	0.2003	0.809	0.002	0.9230	0.984	0.003	0.6310	1.133
D-glucose-6-phosphate	0.000	0.000	-	-	0.000	-	-	0.000	-	-
dihydroxyacetone	0.004	0.003	0.0840	0.832	0.003	0.0430	0.757	0.003	0.1003	0.810
phosphate	0.013	0.006	**0.0014**	**0.492**	0.006	**0.0014**	**0.501**	0.008	0.0117	0.607
D-mannitol	0.003	0.002	0.0738	0.828	0.002	0.0959	0.852	0.002	0.0217	0.704
D-ribose-5-phosphate	0.008	0.005	0.0226	0.684	0.005	0.0134	0.650	0.006	0.0331	0.699
fructose	0.061	0.038	**0.0005**	**0.625**	0.037	**0.0002**	**0.598**	0.036	**0.0003**	**0.581**
fructose-6-phosphate	0.002	0.001	0.0130	0.623	0.002	0.0513	0.716	0.002	0.0642	0.702
fumarate	0.023	0.018	0.0028	0.808	0.019	0.0412	0.861	0.020	0.0724	0.869
gluconate	0.001	0.001	0.0309	0.757	0.001	0.1277	0.834	0.001	0.1221	0.803
glucose	0.541	0.354	**0.0001**	**0.654**	0.429	0.0026	0.793	0.404	0.0620	0.748
glucuronate	0.000	0.000	-	-	0.000	-	-	0.000	-	-
glutamate	2.971	2.134	0.0105	0.718	2.634	0.1499	0.887	2.653	0.1427	0.893
glutamine	0.138	0.070	**<0.0001**	**0.508**	0.087	**0.0005**	**0.630**	0.091	**0.0009**	**0.658**
glycerate	0.000	0.000	-	-	0.000	-	-	0.000	-	-
glycerol	0.047	0.039	0.1451	0.843	0.038	0.1062	0.814	0.039	0.2607	0.847
glycerol 3-phosphate	0.185	0.109	**0.0019**	**0.589**	0.15	0.0628	0.814	0.134	0.0341	0.722
glycine	0.457	0.419	0.0949	0.917	0.427	0.1429	0.935	0.385	0.0332	0.843
glycolate	0.000	0.000	0.7952	0.943	0.000	0.4839	0.882	0.000	0.2418	0.804
glycylglycine	0.015	0.016	0.7353	1.108	0.018	0.5352	1.180	0.017	0.6491	1.146
hexanoate	0.002	0.002	0.5294	0.873	0.002	0.4240	0.839	0.002	0.8874	0.975

Table S2. *Cont.*

Name	MDA-MB-231 Cells									
	Cnormalized = Crelative/Cell Number$_{72\ h}$									
	Control	Genistein			Daidzein			Extract		
		IC20	*p*-Value	FC	IC20	*p*-Value	FC	IC20	*p*-Value	FC
histidine	0.000	0.000	-	-	0.000	-	-	0.000	-	-
hypotaurine	0.045	0.035	0.0292	0.774	0.027	**0.0011**	**0.597**	0.039	0.2057	0.852
indole-2,3-dione	0.000	0.000	-	-	0.000	-	-	0.000	-	-
isocitrate	0.005	0.004	0.0002	0.689	0.004	0.0004	0.704	0.004	0.0008	0.760
isoleucine	0.102	0.092	0.2181	0.906	0.125	0.0508	1.231	0.114	0.1625	1.118
lactate	4.482	3.034	0.0015	0.677	3.322	0.0045	0.741	3.243	0.0105	0.724
leucine	0.167	0.110	**0.0001**	**0.662**	0.149	0.1668	0.892	0.138	0.0248	0.827
lysine	0.008	0.006	0.0260	0.739	0.008	0.4123	0.930	0.007	0.2626	0.881
malate	0.066	0.052	0.0028	0.775	0.053	0.0134	0.800	0.052	0.0042	0.789
methionine	0.027	0.022	0.1356	0.809	0.025	0.2969	0.914	0.022	0.0138	0.798
myo-inositole	6.546	4.757	0.0005	0.727	4.674	0.0003	0.714	5.030	0.0015	0.768
N-acetylaspartate	0.019	0.015	0.0160	0.767	0.011	**0.0004**	**0.551**	0.011	**0.0004**	**0.570**
O-phosphocolamine	0.005	0.003	0.3096	0.694	0.005	0.8274	0.943	0.004	0.4367	0.764
ornithine	0.009	0.010	0.5159	1.051	0.014	0.0002	1.456	0.012	0.0191	1.249
pantothenate	0.004	0.004	0.3814	0.953	0.005	0.2424	1.052	0.004	0.9175	0.984
phosphoenolpyruvate	0.002	0.002	0.0108	0.691	0.002	**0.0051**	**0.645**	0.002	0.0166	0.706
proline	0.428	0.334	0.0086	0.780	0.447	0.5035	1.044	0.450	0.4922	1.052
putrescine	0.127	0.066	**0.0001**	**0.515**	0.069	**0.0001**	**0.545**	0.074	**0.0003**	**0.580**
pyrophosphate	0.375	0.313	0.1555	0.834	0.297	0.1807	0.794	0.381	0.9370	1.018
pyruvate	0.012	0.011	0.1300	0.892	0.011	0.2508	0.884	0.010	0.0154	0.816
ribulose-5-phosphate	0.006	0.004	0.0079	0.696	0.005	0.0755	0.821	0.004	0.0259	0.729
serine	0.140	0.090	**0.0005**	**0.638**	0.122	0.1216	0.871	0.093	**0.0004**	**0.663**
spermidine	0.002	0.001	0.3640	0.763	0.001	0.5213	0.858	0.002	0.8671	1.074
succinate	0.017	0.014	0.0776	0.832	0.011	**0.0011**	**0.665**	0.012	0.0176	0.745
tagatose	0.054	0.035	**0.0006**	**0.654**	0.034	**0.0001**	**0.629**	0.038	0.0012	0.700
threonate	0.028	0.023	0.0100	0.830	0.028	0.6048	0.974	0.027	0.5738	0.956
threonine	0.033	0.027	0.0226	0.845	0.034	0.7579	1.032	0.033	0.8109	1.016
tryptophan	0.009	0.006	0.0082	0.691	0.008	0.2908	0.882	0.007	0.0489	0.827
tyrosine	0.067	0.058	0.1205	0.860	0.067	0.9940	0.996	0.065	0.5871	0.965
urea	0.093	0.076	0.0217	0.817	0.071	0.0029	0.765	0.075	0.0225	0.806
uridine 5′-monophosphate	0.004	0.003	0.3853	0.786	0.003	0.3724	0.763	0.004	0.7781	0.923
valine	0.050	0.029	**0.0019**	**0.586**	0.042	0.1506	0.832	0.037	0.0207	0.740

Table S2. *Cont.*

**Name**	**MDA-MB-231 Cells**									
	**Cnormalized = Crelative/Cell Number$_{72\ h}$**									
	**Control**	**Genistein**			**Daidzein**			**Extract**		
		**IC20**	***p*-Value**	**FC**	**IC20**	***p*-Value**	**FC**	**IC20**	***p*-Value**	**FC**
unknown 1	0.122	0.101	0.1442	0.831	0.106	0.1258	0.869	0.111	0.4321	0.915
unknown 2	0.000	0.000	-	-	0.000	-	-	0.000	-	-

$C_{normalized}$ = The normalized concentration of each metabolite; $C_{relative}$ = The relative concentration after 72 h of treatment; cell number$_{72h}$ = the cell number after 72 h of treatment.
